# Using Life’s Essential 8 and heavy metal exposure to determine infertility risk in American women: a machine learning prediction model based on the SHAP method

**DOI:** 10.3389/fendo.2025.1586828

**Published:** 2025-07-04

**Authors:** Xiaoqing Gu, Qianbing Li, Xiangfei Wang

**Affiliations:** Wuhan Sports University, Wuhan, China

**Keywords:** infertility, Life’s Essential 8, heavy metal exposure, machine learning, SHAP

## Abstract

**Background:**

Fertility status is a marker of future health, and female infertility has been shown to be an important medical and social problem. Life’s Essential 8 (“LE8”) is a comprehensive cardiovascular health assessment proposed by the American Heart Association. The assessment indicators include 4 health behaviors (diet, physical activity, nicotine exposure, and sleep health) and 4 health factors (body mass index, blood lipids, blood glucose, and blood pressure). LE8 and heavy metal exposure have both been shown to be associated with infertility. However, the association between LE8 and heavy metal exposure and female infertility has not been investigated. The aim of this study was to develop a machine learning prediction model for LE8 and heavy metal exposure and the risk of female infertility in the United States.

**Methods:**

The National Health and Nutrition Examination Survey (“NHANES”) is a nationally representative program conducted by the National Center for Health Statistics to assess the health and nutritional status of the U.S. population. For this study, 873 women between the ages of 20 and 45 were selected from the 2013–2018 NHANES dataset. The association between LE8 and heavy metal exposure and risk of infertility was assessed using logistic regression analysis and six machine learning models (Decision Tree, GBDT, AdaBoost, LGBM, Logistic Regression, Random Forest), and the SHAP algorithm was used to explain the model’s decision process.

**Results:**

Of the six machine learning models, the LGBM model has the best predictive performance, with an AUROC of 0.964 on the test set. SHAP analysis showed that LE8, body mass index (“BMI”), diet, Cadmium (“Cd”), Cesium (“Cs”), Molybdenum (“Mo”), Antimony (“Sb”), Tin (“Sn”), education level and pregnancy history were significantly associated with the risk of female infertility. Cd, BMI and LE8 are the variables that contribute most to the prediction of infertility risk. Among them, BMI and LE8 have a negative predictive effect on female infertility in the model, while Cd has a positive contribution to the prediction of female infertility. Further analysis showed that there was a significant interaction between heavy metals and LE8, which may have a synergistic effect on the risk of female infertility.

**Conclusions:**

This study used LE8 and heavy metal exposure to create a machine learning model that predicts the risk of female infertility. The model identified ten key factors. The model demonstrated high predictive accuracy and good clinical interpretability. In the future, LE8 and heavy metal exposure can be used to screen for female infertility early on.

## 1 Introduction

Female infertility is the failure to achieve pregnancy after 12 months or more of regular unprotected sex with the same sexual partner in women of childbearing age (1). The World Health Organization (WHO) has recognized infertility as a global public health problem. Approximately 15% of couples of reproductive age worldwide are affected by infertility. Among these, the infertility rate among women of reproductive age in the United States is approximately 15.5% and is increasing at a rate of 0.37% per year (2). Common causes of female infertility include male factor infertility (3), endometriosis (4), and fallopian tube damage (5). As society modernizes, the impact of external environmental and personal health factors on women’s reproductive health cannot be ignored. Exposure to environmental factors, unhealthy lifestyles (such as smoking and sedentary lifestyles), and obesity-related metabolic disorders can also increase the risk of infertility in women (6, 7). According to statistics from the NHANES, nearly every pregnant U.S. woman is exposed to at least 43 different potentially harmful chemicals during her pregnancy (8). Female infertility is not only a huge financial burden for patients and the healthcare system, it can also lead to an increase in mental disorders such as depression and anxiety in women (9). In addition, a number of studies have suggested that infertile women may be at increased risk for gynecologic malignancies (10–12). Predicting and identifying risk factors for female infertility is important for restoring the overall fertility rate in the United States, reducing the medical and economic burden, and optimizing the allocation of healthcare resources. Currently, most diagnoses of female infertility are made using imaging tests such as Hysterosalpingography, Magnetic Resonance Imaging and Computed Tomography. Although these examinations can provide professional and effective pathological diagnoses, a comprehensive assessment of the causes of infertility usually requires a combination of multiple examinations, which places high demands on equipment and technology. In addition, the cost of the examination is relatively high, which cannot meet the needs of universal medical care for a wider range of people.

LE8 is a comprehensive cardiovascular health assessment proposed by the American Heart Association. The assessment indicators include 4 health behaviors and 4 health factors, namely diet, physical activity, nicotine exposure, and sleep health, BMI, blood lipids, blood glucose, and blood pressure (13). Currently, researchers are using the LE8 score for risk prediction studies of all-cause mortality risk (14–16), adult cardiovascular health (17, 18), and cardiovascular disease risk (19, 20). Previous studies have applied the LE8 score to research on female infertility, based on its association with cardiovascular health (21, 22). The results indicate that various LE8 indicators are associated with female infertility. Polycystic ovary syndrome is one of the primary causes of female infertility and exhibits complex interactions with obesity. This provides further evidence of the significant association between obesity and female infertility (23). Studies have shown that women with a BMI over 25 kg/m² before pregnancy are more likely to have difficulty conceiving than women with an ideal BMI (between 20 and 24.99 kg/m²) (24). Specifically, the impact of obesity on female fertility involves physiological, endocrine, and metabolic factors (25), such as abnormal high-density lipoprotein metabolism and elevated levels of unesterified cholesterol. These factors may interfere with oocyte meiosis arrest and developmental potential, thereby causing female infertility (26). Not only is physical activity an effective way to lose weight, but it can also improve reproductive function in women with polycystic ovary syndrome. This includes increasing menstrual regularity and ovulation rate, improving sexual function, and reducing infertility (27). A weight reduction of 2.5% to 5% has been demonstrated to enhance blood sugar, triglycerides, and other indicators (28), good blood sugar control has been shown to possess the potential to improve reproductive health (22). Consequently, interventions such as the adoption of a low-GI diet or a Mediterranean diet have been shown to be effective in the treatment of polycystic ovary syndrome and the promotion of reproductive health (29). Furthermore, extant research has demonstrated a significant correlation between sleep disturbances, tobacco use, and elevated blood pressure with female infertility. Sleep disorders are more prevalent among women experiencing fertility issues, primarily influenced by mechanisms such as disruption of circadian rhythms, abnormalities in melatonin and hormone regulation, and oxidative stress (30). The correlation between smoking and infertility risk is particularly salient among Mexican Americans and women aged 25 to 38 years (31). In women under the age of 30, infertility has been observed to be associated with elevated systolic and diastolic blood pressure. However, no such association has been demonstrated in older women. Consequently, the monitoring of weight and blood pressure in women with a history of infertility is of particular importance in clinical practice (32). In light of the aforementioned research results, it has been determined that all factors included in the LE8 score exert varying degrees of influence on the incidence of female infertility. The LE8 score offers a novel approach to the screening of female infertility by indirectly predicting the risk of female infertility.

Heavy metals are defined as inorganic elements that possess a density greater than 5 g/cm3 (33). Heavy metals are divided into two groups based on their toxicity: essential and non-essential. Essential heavy metals such as Zn, Cu, Fe and Co are harmless or relatively harmless at low concentrations, while non-essential heavy metals such as Cd, Pb, Hg, As and Cr are highly toxic even at low concentrations (34). The mechanism of health effects induced by oxidative stress after heavy metal exposure (35)has been demonstrated to increase the risk of diseases such as neurological diseases (36), cardiovascular diseases (37), immune system diseases (38), and metabolic syndrome (39). Nevertheless, the marked rise in environmental pollutants over the past few decades has resulted in deleterious physiological effects. Heavy metals are among the most significant pollutants impacting our environment (40). Previous studies have shown an association between heavy metal exposure and female infertility. Increased exposure to environmental heavy metals may affect the regulation and function of the female reproductive system, such as causing polycystic ovary syndrome in women (41), which in turn may increase the risk of female infertility (42). Furthermore, the findings of numerous studies have demonstrated a negative correlation between the presence of Cu, Cr, Co, Cd, Rb, Hg, and Pb and fertility levels. For instance, Hg levels that exceed safe thresholds have been observed to disrupt menstrual cycles in women, potentially contributing to infertility (43, 44). Heavy metals can accumulate in the blood, urine, liver, and other tissues of the human body and adversely affect the body. An earlier cross-sectional study indicated that the blood levels of Cd and Pb in U.S. women were positively correlated with infertility (45). Subsequent studies have found that urine may better reflect the reproductive toxicity of long-term heavy metal exposure than blood, and have confirmed that urinary As and Cd are potential risk factors for female infertility. Urinary As is significantly and positively correlated with female infertility, and Pb exposure has a more pronounced effect on the risk of infertility in older (35–44 years) and obese (BMI≧30 kg/m²) women (46). Based on the above research, we found that independent exposure to heavy metals is significantly associated with the risk of female infertility, and the indirect prediction of the risk of female infertility by the content of a single heavy metal in women’s urine may provide new opportunities for the initial screening of female infertility.

Recent research has discussed the relationship between environmental exposure and the combined effect of LE8 on cardiovascular health (47), liver disease (48), and increased risk of death (49). Due to advancements in exposomics testing technologies and individual differences in reproductive health, such as endocrine metabolic heterogeneity and ovarian reserve function, the relationship between various LE8 indicators and heavy metal exposure, as well as their potential mechanisms related to infertility risk, remains unclear. The extant literature on the subject is predominantly confined to linear association analyses of individual factors and reproductive health. There is a paucity of systematic exploration of the synergistic effects of multiple exposure factors and the construction of predictive models. The incorporation of LE8 and heavy metal exposure into risk prediction models has the potential to provide a more comprehensive assessment of the multidimensional causes of female infertility. In principle, this broadens the scope of single-dimensional analysis, while methodologically improving the accuracy and adaptability of prediction models. Consequently, this demonstrates significant practical value.

In the course of our investigation into the nonlinear associations and temporal interactions between LE8, multiple biological indicators, and heavy metal exposure factors, it became evident that traditional linear regression models are encumbered by significant limitations, particularly when confronted with multiple collinearity or confounding factors in the data. These limitations can readily result in model overfitting and a decline in predictive performance. Machine learning (ML) has demonstrated remarkable proficiency in the identification and analysis of intricate correlation patterns within high-dimensional data sets. The program’s advanced feature selection and nonlinear modeling capabilities enable the identification of potential variable interaction effects and non-explicit feature structures. At present, the application has been successfully implemented in the prediction of disease risks, including coronary artery disease (50) and male infertility (51). SHapley Additive exPlanations (SHAP) is an advanced visualization technique designed to improve the interpretability of ML decision making. It significantly overcomes the limitations of the traditional “black box” nature of the model by intuitively quantifying the marginal contribution of each feature to the model prediction (52, 53). Furthermore, the integration of model outputs with epidemiological and toxicological evidence pertaining to female infertility has the potential to enhance the interpretability and biological plausibility of research findings, thereby underscoring the methodological potential of interpretable machine learning in studies investigating female reproductive health and infertility risk prediction. NHANES is a nationally representative program of the National Center for Health Statistics (NCHS) that assesses the health and nutritional status of the U.S. population (54). The database integrates input from global experts across over 400 domains, covering demographic information, dietary intake, physical examinations, laboratory measurements, and questionnaire data. To ensure national representativeness, NHANES applies complex statistical methodologies, including stratified and multistage probability sampling.

Therefore, the present study utilized a substantial sample population from the NHANES (2013-2018) as a foundation, incorporated LE8 and heavy metal exposure indicators, and constructed a composite risk prediction model for female infertility. The specific objectives of this study are as follows: (1) The identification of key variables influencing infertility in LE8 and indicators of heavy metal exposure is imperative. (2) The utilization of multiple machine learning algorithms, including GBDT, AdaBoost, and LGBM, in conjunction with the SHAP explainability framework, enabled the development of a risk prediction model for female infertility that is both clinically interpretable and actionable. (3) The present study aims to elucidate the interactive mechanisms between lifestyle and environmental exposure, thereby providing a theoretical basis and methodological support for the early identification and intervention of female infertility risk.

## 2 Methods

### 2.1 Research subjects

**Figure 1:**
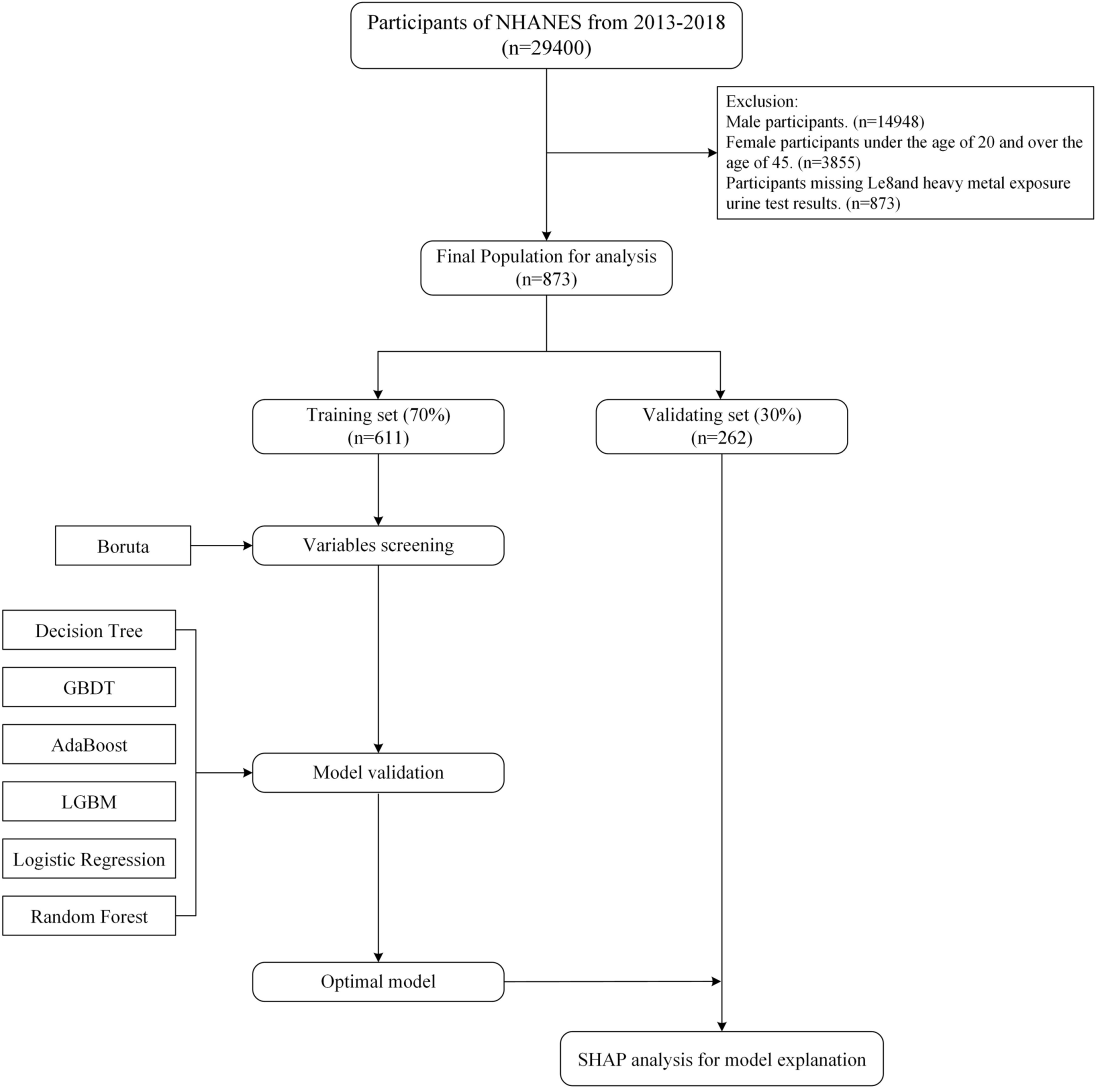
Flow chart of sample selection from the NHANES 2013–2018.

The NHANES study was approved by the NCHS Ethics Committee, which obtained written informed consent from each participant. All NHANES data are openly accessible and have been de-identified (https://www.cdc.gov/nchs/nhanes/about/erb.html). NHANES only measured heavy metal exposure in urine from 2013 to 2018. Therefore, this study used data from this database from 2013 to 2018. The study focused on female participants between the ages of 20 and 45. From the 29,400 participants in the 2013–2018 cycle, the study excluded male participants (n=14,452), female participants younger than 20 years and older than 45 years (n=11,093), and participants with missing or incomplete LE8 indicator and heavy metal exposure urine test results (n=2,982). Missing data for all other variables was less than 20%, so multiple imputation was used to fill in the missing values. The final sample size was 873 female participants. The specific screening and research process is shown in **Figure 1**.

### 2.2 Assessment of infertility

The present study employs female infertility as the primary outcome variable. In accordance with the prevailing clinical definition of infertility, which is defined as “a reproductive-age woman who has not conceived after 12 months or more of regular unprotected sexual intercourse with the same sexual partner” (1), the present study employs items from the Reproductive Health Questionnaire (RHQ) in the NHANES database that are highly consistent with this definition. “{Have you/Has SP} ever attempted to become pregnant over a period of at least a year without becoming pregnant?” Infertile respondents were defined as those who answered “yes” to the aforementioned question, while fertile respondents were defined as those who answered “no”. The data were collected by professional interviewers using a computer-assisted personal interview standard procedure, ensuring high consistency and reliability. The relevant methods have been widely applied in reproductive epidemiological studies.

### 2.3 Review of LE8

The LE8 scoring algorithm encompasses four categories of healthy behaviors (diet, physical activity, nicotine exposure, and sleep health) and four categories of health factors (BMI, blood lipids, blood glucose, and blood pressure). The eight cardiovascular health (CVH) indicators are scored in accordance with the American Heart Association’s established criteria, with a scale ranging from 0 to 100 points for each indicator. Scores ranging from 0 to 49 are classified as low level, 50 to 79 are considered moderate level, and 80 to 100 are designated as high level (13). The LE8 score is the arithmetic mean of these eight indicators.

### 2.4 Evaluation of heavy metal exposure

In the present study, an exhaustive analysis was conducted on all heavy metals detectable in urine samples from participants in the NHANES database, a total of 11 heavy metals: Barium (Ba), Cadmium (Cd), Cobalt (Co), Cesium (Cs), Manganese (Mn), Molybdenum (Mo), Lead (Pb), Antimony (Sb), Tin (Sn), Thallium (Tl), Titanium (Tu). All measurements were performed using Inductively Coupled Plasma Mass Spectrometry (ICP-DRCMS) at the National Environmental Health Laboratory, ensuring the accuracy of the heavy metal exposure data and providing an important basis for the subsequent relationship between heavy metal exposure and female infertility rates. Further information regarding standard laboratory procedures can be found on the NHANES website.

### 2.5 Covariate

Covariates included age, ethnicity, education level, work situation, pregnancy history, and chronic disease status. Among them, age is a continuous variable and the remaining 5 items are categorical variables that need to be processed numerically. The assignment of the ethnicity variable is divided into five levels corresponding to Mexican American, Other Hispanic, Non-Hispanic White, Non-Hispanic Black, and Other Ethnic Group - Including Multiracial, from 1 to 5. Assign values from 1 to 6 to the work statuses an employee of a private company, business, or individual for wages, salary, or commission; a federal government employee; a state government employee; a local government employee; self-employed in own business, professional practice or farm; working without pay in family business or farm, respectively. The education level variable is assigned a value from 1 to 5, corresponding to less than 9th grade, 9th-11th grade (including 12th grade without a diploma), high school graduate/GED or equivalent, some college or AA degree, and college graduate or higher. The pregnancy history variable assigns a value of 1 to those who have ever been pregnant and 0 to those who have never been pregnant, and the chronic disease status variable assigns a value of 1 to those who have a chronic disease and 0 to those who do not. These covariates were selected based on their established relevance in reproductive medicine research and their potential confounding effect on research results.

### 2.6 Statistical analysis

#### 2.6.1 Baseline regression analysis and logistic regression analysis

In the baseline analysis, the demographic characteristics are expressed as the median and its interquartile range (IQR), while the categorical variables are expressed as counts and percentages. The heavy metal factor was transformed by natural logarithm (log10) and further divided into four parts (Q1-Q4). The Pearson correlation coefficient was used to evaluate the relationship between the levels of 11 heavy metals. The overall score and scores for the eight dimensions in LE8 were assigned values of Q1, Q2, and Q3 according to low, medium, and high levels, respectively. In order to preliminarily assess the association between heavy metal exposure, LE8, and female infertility, univariate logistic regression was used. Among these factors, heavy metal exposure and LE8 factors were analyzed using continuous (log-transformed) and categorical variables. Heavy metal exposure factors were divided into four quartiles (55), and LE8 factors were divided into three tertiles (13), with Q1 as the reference category. This approach was used to preliminarily assess the association between these factors and infertility. Concurrently, categorical variables were converted to integer values p for trend tests. To control for confounding factors, two models were constructed for analysis: one unadjusted for any covariates, and another adjusted for age, ethnicity, education level, employment status, pregnancy history, and chronic disease.

#### 2.6.2 Model development

This study employs the general classification method, which involves the division of data into a training set comprising 70% of the total and a test set constituting the remaining 30% (56). First, based on the training set and test set, and considering that high-dimensional complex data may lead to classification performance degradation and overfitting in machine learning algorithms, the Boruta feature selection algorithm based on random forest classifiers is introduced before constructing the machine learning model to assess the feature importance of LE8, heavy metals, and other confounding factors, thereby screening out the most critical variables. The Boruta algorithm is a feature selection method based on random forests that can identify the most important features related to the dependent variable in a data set through multiple dynamic iterations (57).

Secondly, a combination of Synthetic Minority Over-sampling Technique (SMOTE) and undersampling techniques was employed to achieve a balanced distribution between infertile and non-infertile populations. Subsequently, six machine learning methods—Decision Tree, GBDT, AdaBoost, LGBM, Logistic Regression, and Random Forest—were employed to construct models for the core variables to assess the predictive ability of LE8 and heavy metal exposure on female infertility. In the context of model validation, a range of metrics, including Accuracy, Recall, F1 score, and the Matthews Correlation Coefficient (MCC), were employed to assess the predictive capabilities of six machine learning (ML) models. The objective of this assessment was to identify the ML model that demonstrated the most optimal predictive performance. The range of AUROC values is from 0.5 to 1.0 (58). It is evident that as the value within this range increases, the predictive ability concomitantly improves. Following the selection of the optimal model, the overall performance of the model is evaluated using the K-fold cross-validation method. In this study, we employed one part of K as the test set, repeated the model establishment and evaluation cycles K times, and calculated the average (59). This approach has been demonstrated to effectively mitigate performance estimation errors arising from disparate data partitioning methodologies, thereby ensuring a more stable and reliable model performance evaluation.

In addition, the SHAP algorithm is employed to elucidate the decision-making process of the machine learning model, as predicted by the optimal model. The specific contribution of each feature to the final prediction is detailed through its Shapley value and presented visually. Subsequently, through subgroup interaction analysis, we further explored the interaction between heavy metals and the various dimensions of LE8. In the specific analysis process, each factor was analyzed using categorical variables, and confounding factors were controlled. Finally, to further assess the robustness of the model results and the potential impact of confounding biases, this study employed propensity score matching (PSM) for sensitivity analysis. Using infertility as the matching criterion, propensity scores were calculated based on LE8, heavy metal exposure, and covariates. A 1:8 nearest neighbor matching method was employed to construct a matched sample set with balanced characteristics. The construction of the model and the subsequent evaluation of its predictive performance were repeated on the matched samples. This process was undertaken to assess the predictive stability of the original model under various sample structures.

## 3 Results

### 3.1 Baseline characteristics

A total of 103 infertile patients were selected for baseline analysis compared to 770 individuals without infertility, and baseline characteristics are shown in **Tables 1**–**3**. Among the 873 participants in this study (mean age: 33.10), the infertility group had a higher mean age (35.09 vs. 32.84, *p*<0.05). The two groups exhibited significant differences in age, pregnancy history, prevalence of chronic diseases (asthma, overweight, and arthritis), heavy metal exposure (cadmium), and LE8-related indicators (LE8, BMI, sleep health, and blood pressure) (*p*<0.05).

**Table 1:** Comparison of Covariates between Infertility and Non-infertility groups.

Variable	Overall	None Infertility	Infertility	*p*-value
	N = 873	N = 770	N = 103	
Age	33.10 (7.52)	32.84 (7.54)	35.09 (7.14)	0.003
Ethnicity				0.573
Mexican American	164 (18.79%)	140 (18.18%)	24 (23.30%)	
Other Hispanic	90 (10.31%)	83 (10.78%)	7 (6.80%)	
Non-Hispanic White	297 (34.02%)	264 (34.29%)	33 (32.04%)	
Non-Hispanic Black	175 (20.05%)	153 (19.87%)	22 (21.36%)	
Other Ethnic Group - Including Multiracial	147 (16.84%)	130 (16.88%)	17 (16.50%)	
Education				0.643
Less than 9th grade	40 (4.58%)	36 (4.68%)	4 (3.88%)	
9-11th grade (Includes 12th grade with no diploma)	89 (10.19%)	79 (10.26%)	10 (9.71%)	
High school graduate/GED or equivalent	182 (20.85%)	166 (21.56%)	16 (15.53%)	
Some college or AA degree	326 (37.34%)	284 (36.88%)	42 (40.78%)	
College graduate or above	236 (27.03%)	205 (26.62%)	31 (30.10%)	
Work situation				0.825
An employee of a private company, business, or individual for wages, salary, or commission	745 (85.34%)	657 (85.32%)	88 (85.44%)	
A federal government employee	9 (1.03%)	9 (1.17%)	0 (0.00%)	
A state government employee	52 (5.96%)	46 (5.97%)	6 (5.83%)	
A local government employee	40 (4.58%)	35 (4.55%)	5 (4.85%)	
A state government employee	24 (2.75%)	20 (2.60%)	4 (3.88%)	
Working without pay in family business or farm	3 (0.34%)	3 (0.39%)	0 (0.00%)	
Ever pregnant				0.003
No	225 (25.77%)	211 (27.40%)	14 (13.59%)	
Yes	648 (74.23%)	559 (72.60%)	89 (86.41%)	
Hypertension				0.103
No	742 (84.99%)	660 (85.71%)	82 (79.61%)	
Yes	131 (15.01%)	110 (14.29%)	21 (20.39%)	
Diabetes				0.284
No	832 (95.30%)	736 (95.58%)	96 (93.20%)	
Yes	41 (4.70%)	34 (4.42%)	7 (6.80%)	
Asthma				<0.001
No	711 (81.44%)	640 (83.12%)	71 (68.93%)	
Yes	162 (18.56%)	130 (16.88%)	32 (31.07%)	
Overweight				0.004
No	504 (57.73%)	458 (59.48%)	46 (44.66%)	
Yes	369 (42.27%)	312 (40.52%)	57 (55.34%)	
Arthritis				0.030
No	789 (90.38%)	702 (91.17%)	87 (84.47%)	
Yes	84 (9.62%)	68 (8.83%)	16 (15.53%)	
Heart failure				0.463
No	869 (99.54%)	766 (99.48%)	103 (100.00%)	
Yes	4 (0.46%)	4 (0.52%)	0 (0.00%)	
Coronary heart disease				0.247
No	870 (99.66%)	768 (99.74%)	102 (99.03%)	
Yes	3 (0.34%)	2 (0.26%)	1 (0.97%)	
Anginapectoris				0.711
No	867 (99.31%)	765 (99.35%)	102 (99.03%)	
Yes	6 (0.69%)	5 (0.65%)	1 (0.97%)	
Heart attack				0.463
No	869 (99.54%)	766 (99.48%)	103 (100.00%)	
Yes	4 (0.46%)	4 (0.52%)	0 (0.00%)	
Stroke				0.412
No	868 (99.43%)	765 (99.35%)	103 (100.00%)	
Yes	5 (0.57%)	5 (0.65%)	0 (0.00%)	
Emphysema				0.463
No	869 (99.54%)	766 (99.48%)	103 (100.00%)	
Yes	4 (0.46%)	4 (0.52%)	0 (0.00%)	
Chronic bronchitis				0.520
No	833 (95.42%)	736 (95.58%)	97 (94.17%)	
Yes	40 (4.58%)	34 (4.42%)	6 (5.83%)	
Liver condition				0.140
No	857 (98.17%)	754 (97.92%)	103 (100.00%)	
Yes	16 (1.83%)	16 (2.08%)	0 (0.00%)	
Chronic disease				<0.001
No	366 (41.92%)	339 (44.03%)	27 (26.21%)	
Yes (one kind)	281 (32.19%)	246 (31.95%)	35 (33.98%)	
Yes (two or more)	226 (25.89%)	185 (24.03%)	41 (39.81%)	

**Table 2:** Comparison of LE8 between Infertility and Non-infertility groups.

Variable	Overall	None Infertility	Infertility	*p*-value
	N = 873	N = 770	N = 103	
LE8				0.002
49≥x≥0	69 (7.90%)	52 (6.75%)	17 (16.50%)	
79≥x≥50	409 (46.85%)	363 (47.14%)	46 (44.66%)	
100≥x≥80	395 (45.25%)	355 (46.10%)	40 (38.83%)	
Diet				0.659
49≥x≥0	458 (52.46%)	400 (51.95%)	58 (56.31%)	
79≥x≥50	210 (24.05%)	186 (24.16%)	24 (23.30%)	
100≥x≥80	205 (23.48%)	184 (23.90%)	21 (20.39%)	
Physical activity				0.179
49≥x≥0	213 (24.40%)	185 (24.03%)	28 (27.18%)	
79≥x≥50	38 (4.35%)	37 (4.81%)	1 (0.97%)	
100≥x≥80	622 (71.25%)	548 (71.17%)	74 (71.84%)	
Nicotine exposure				0.420
49≥x≥0	176 (20.16%)	152 (19.74%)	24 (23.30%)	
79≥x≥50	34 (3.89%)	32 (4.16%)	2 (1.94%)	
100≥x≥80	663 (75.95%)	586 (76.10%)	77 (74.76%)	
Sleep health				0.031
49≥x≥0	155 (17.75%)	131 (17.01%)	24 (23.30%)	
79≥x≥50	135 (15.46%)	113 (14.68%)	22 (21.36%)	
100≥x≥80	583 (66.78%)	526 (68.31%)	57 (55.34%)	
BMI				<0.001
49≥x≥0	393 (45.02%)	327 (42.47%)	66 (64.08%)	
79≥x≥50	211 (24.17%)	199 (25.84%)	12 (11.65%)	
100≥x≥80	269 (30.81%)	244 (31.69%)	25 (24.27%)	
Blood lipids				0.544
49≥x≥0	136 (15.58%)	118 (15.32%)	18 (17.48%)	
79≥x≥50	202 (23.14%)	175 (22.73%)	27 (26.21%)	
100≥x≥80	535 (61.28%)	477 (61.95%)	58 (56.31%)	
Blood glucose				0.446
49≥x≥0	39 (4.47%)	32 (4.16%)	7 (6.80%)	
79≥x≥50	95 (10.88%)	83 (10.78%)	12 (11.65%)	
100≥x≥80	739 (84.65%)	655 (85.06%)	84 (81.55%)	
Blood pressure				0.004
49≥x≥0	61 (6.99%)	46 (5.97%)	15 (14.56%)	
79≥x≥50	115 (13.17%)	100 (12.99%)	15 (14.56%)	
100≥x≥80	697 (79.84%)	624 (81.04%)	73 (70.87%)	

**Table 3:** Comparison of Heavy Metals between Infertility and Non-infertility groups.

Variable	Overall	None Infertility	Infertility	*p*-value
	N = 873	N = 770	N = 103	
Ba				0.591
Q1	218 (24.97%)	196 (25.45%)	22 (21.36%)	
Q2	218 (24.97%)	194 (25.19%)	24 (23.30%)	
Q3	218 (24.97%)	192 (24.94%)	26 (25.24%)	
Q4	219 (25.09%)	188 (24.42%)	31 (30.10%)	
Cd				0.017
Q1	218 (24.97%)	198 (25.71%)	20 (19.42%)	
Q2	218 (24.97%)	201 (26.10%)	17 (16.50%)	
Q3	218 (24.97%)	182 (23.64%)	36 (34.95%)	
Q4	219 (25.09%)	189 (24.55%)	30 (29.13%)	
Co				0.433
Q1	218 (24.97%)	199 (25.84%)	19 (18.45%)	
Q2	218 (24.97%)	191 (24.81%)	27 (26.21%)	
Q3	218 (24.97%)	189 (24.55%)	29 (28.16%)	
Q4	219 (25.09%)	191 (24.81%)	28 (27.18%)	
Cs				0.804
Q1	218 (24.97%)	195 (25.32%)	23 (22.33%)	
Q2	218 (24.97%)	194 (25.19%)	24 (23.30%)	
Q3	218 (24.97%)	189 (24.55%)	29 (28.16%)	
Q4	219 (25.09%)	192 (24.94%)	27 (26.21%)	
Mn				0.505
Q1	218 (24.97%)	193 (25.06%)	25 (24.27%)	
Q2	218 (24.97%)	195 (25.32%)	23 (22.33%)	
Q3	218 (24.97%)	195 (25.32%)	23 (22.33%)	
Q4	219 (25.09%)	187 (24.29%)	32 (31.07%)	
Mo				0.942
Q1	218 (24.97%)	194 (25.19%)	24 (23.30%)	
Q2	218 (24.97%)	192 (24.94%)	26 (25.24%)	
Q3	218 (24.97%)	190 (24.68%)	28 (27.18%)	
Q4	219 (25.09%)	194 (25.19%)	25 (24.27%)	
Pb				0.828
Q1	218 (24.97%)	194 (25.19%)	24 (23.30%)	
Q2	218 (24.97%)	195 (25.32%)	23 (22.33%)	
Q3	218 (24.97%)	190 (24.68%)	28 (27.18%)	
Q4	219 (25.09%)	191 (24.81%)	28 (27.18%)	
Sb				0.484
Q1	218 (24.97%)	198 (25.71%)	20 (19.42%)	
Q2	218 (24.97%)	193 (25.06%)	25 (24.27%)	
Q3	218 (24.97%)	190 (24.68%)	28 (27.18%)	
Q4	219 (25.09%)	189 (24.55%)	30 (29.13%)	
Sn				0.719
Q1	218 (24.97%)	191 (24.81%)	27 (26.21%)	
Q2	218 (24.97%)	197 (25.58%)	21 (20.39%)	
Q3	218 (24.97%)	190 (24.68%)	28 (27.18%)	
Q4	219 (25.09%)	192 (24.94%)	27 (26.21%)	
Tl				0.505
Q1	218 (24.97%)	195 (25.32%)	23 (22.33%)	
Q2	218 (24.97%)	193 (25.06%)	25 (24.27%)	
Q3	218 (24.97%)	195 (25.32%)	23 (22.33%)	
Q4	219 (25.09%)	187 (24.29%)	32 (31.07%)	
Tu				0.696
Q1	218 (24.97%)	190 (24.68%)	28 (27.18%)	
Q2	218 (24.97%)	197 (25.58%)	21 (20.39%)	
Q3	218 (24.97%)	190 (24.68%)	28 (27.18%)	
Q4	219 (25.09%)	193 (25.06%)	26 (25.24%)	

### 3.2 Logistic regression

The results of logistic regression analysis (**Tables 4**, **5**) show that LE8, BMI, sleep health, blood pressure and heavy metal Cd are correlated with female infertility through multiple model validation. In Model 1, which did not adjust for covariates, LE8, BMI, blood pressure, sleep health, and heavy metal Cd were found to be significantly associated with the risk of female infertility when considered continuous variables. In Model 2, which was adjusted for age, ethnicity, education level, employment status, pregnancy history, and five chronic diseases as covariates, only LE8, BMI, blood pressure, and sleep health remained significantly associated with the risk of female infertility. As categorical variables, in model 1, LE8 (Q2, Q3), BMI (Q2, Q3), blood pressure (Q3), sleep health (Q3), and heavy metal Cd (Q3) were significantly associated with the risk of female infertility. LE8 (Q2, Q3), BMI (Q2, Q3), blood pressure (Q2, Q3), and sleep health (Q3) in model 2 were associated with the risk of female infertility.

**Table 4:** Correlation between LE8 and Infertility.

Variable	Model1		Model2	
	OR(95%CI)	Pvalue	OR(95%CI)	*p*-value
LE8	0.977(0.963-0.990)	0.001	0.971(0.955-0.986)	0.000
Q1				
Q2	0.388(0.210-0.741)	0.003	0.354(0.185-0.694)	0.002
Q3	0.345(0.184-0.664)	0.001	0.293(0.145-0.605)	0.001
P for trend		0.010		0.005
BMI	0.990(0.984-0.995)	0.000	0.989(0.983-0.995)	0.001
Q1				
Q2	0.299(0.150-0.547)	0.000	0.278(0.138-0.518)	0.000
Q3	0.508(0.306-0.818)	0.007	0.492(0.283-0.832)	0.010
P for trend		0.001		0.002
Blood pressure	0.988(0.981-0.996)	0.003	0.988(0.980-0.997)	0.007
Q1				
Q2	0.460(0.206-1.024)	0.056	0.397(0.172-0.913)	0.029
Q3	0.359(0.195-0.693)	0.001	0.331(0.166-0.681)	0.002
P for trend		0.003		0.005
Blood glucose	0.994(0.984-1.005)	0.274	0.996(0.986-1.008)	0.520
Q1				
Q2	0.661(0.243-1.913)	0.425	0.783(0.279-2.327)	0.646
Q3	0.586(0.265-1.484)	0.218	0.723(0.313-1.888)	0.473
P for trend		0.241		0.489
Blood lipids	0.995(0.988-1.003)	0.185	0.996(0.989-1.004)	0.324
Q1				
Q2	1.011(0.537-1.946)	0.972	0.997(0.522-1.940)	0.992
Q3	0.797(0.461-1.438)	0.432	0.841(0.478-1.542)	0.561
P for trend		0.322		0.476
Diet	0.997(0.990-1.004)	0.385	0.996(0.989-1.003)	0.241
Q1				
Q2	0.890(0.528-1.460)	0.652	0.826(0.483-1.375)	0.471
Q3	0.787(0.455-1.316)	0.375	0.729(0.409-1.258)	0.268
P for trend		0.362		0.243
Physical activity	0.998(0.994-1.003)	0.522	0.998(0.993-1.004)	0.515
Q1				
Q2	0.179(0.010-0.879)	0.096	0.161(0.009-0.805)	0.079
Q3	0.892(0.566-1.441)	0.631	0.884(0.552-1.450)	0.617
P for trend		0.783		0.787
Sleep health	0.991(0.983-0.999)	0.017	0.989(0.981-0.997)	0.006
Q1				
Q2	1.063(0.563-1.999)	0.850	0.815(0.420-1.572)	0.542
Q3	0.591(0.358-1.004)	0.045	0.498(0.290-0.872)	0.013
P for trend		0.019		0.007
Nicotine exposure	0.998(0.993-1.003)	0.464	0.996(0.990-1.002)	0.194
Q1				
Q2	0.396(0.062-1.428)	0.223	0.321(0.049-1.199)	0.142
Q3	0.832(0.516-1.385)	0.464	0.687(0.398-1.209)	0.183
P for trend		0.562		0.251

**Table 5:** Correlation between Heavy metal exposure and Infertility.

Variable	Model1		Model2	
	OR(95%CI)	Pvalue	OR(95%CI)	*p*-value
Ba	1.287(0.827-2.005)	0.263	1.349(0.857-2.127)	0.196
Q1				
Q2	1.051(0.565-1.957)	0.875	1.139(0.608-2.142)	0.684
Q3	1.259(0.694-2.307)	0.449	1.352(0.730-2.527)	0.338
Q4	1.469(0.825-2.656)	0.195	1.566(0.865-2.878)	0.142
P for trend		0.152		0.115
Cd	1.603(1.009-2.561)	0.047	1.386(0.842-2.295)	0.201
Q1				
Q2	0.743(0.371-1.460)	0.392	0.788(0.391-1.571)	0.499
Q3	1.856(1.053-3.343)	0.035	1.752(0.962-3.267)	0.071
Q4	1.489(0.828-2.723)	0.188	1.325(0.701-2.544)	0.390
P for trend		0.029		0.110
Co	1.172(0.693-1.990)	0.555	1.138(0.668-1.948)	0.636
Q1				
Q2	1.481(0.801-2.786)	0.214	1.584(0.844-3.027)	0.156
Q3	1.607(0.878-3.005)	0.129	1.607(0.866-3.044)	0.137
Q4	1.535(0.835-2.880)	0.172	1.550(0.825-2.966)	0.177
P for trend		0.180		0.207
Cs	1.047(0.550-2.015)	0.890	0.948(0.496-1.834)	0.873
Q1				
Q2	0.953(0.518-1.751)	0.877	1.117(0.603-2.076)	0.723
Q3	1.240(0.698-2.223)	0.464	1.304(0.720-2.385)	0.383
Q4	1.137(0.633-2.052)	0.668	1.109(0.605-2.045)	0.738
P for trend		0.492		0.645
Mn	1.452(0.612-3.097)	0.360	1.412(0.579-3.112)	0.415
Q1				
Q2	1.206(0.662-2.218)	0.541	0.854(0.460-1.578)	0.614
Q3	1.154(0.629-2.130)	0.643	0.901(0.485-1.666)	0.738
Q4	1.414(0.791-2.564)	0.246	1.279(0.722-2.288)	0.401
P for trend		0.289		0.366
Mo	1.145(0.693-1.909)	0.599	1.164(0.698-1.961)	0.563
Q1				
Q2	1.000(0.553-1.808)	1.000	1.095(0.600-2.005)	0.767
Q3	1.138(0.640-2.033)	0.660	1.233(0.680-2.253)	0.491
Q4	0.995(0.550-1.798)	0.986	1.023(0.555-1.890)	0.942
P for trend		0.901		0.850
Pb	1.262(0.748-2.130)	0.383	1.221(0.715-2.088)	0.464
Q1				
Q2	1.049(0.571-1.930)	0.877	0.945(0.508-1.756)	0.858
Q3	1.249(0.696-2.263)	0.457	1.207(0.665-2.206)	0.536
Q4	1.243(0.692-2.251)	0.467	1.123(0.617-2.058)	0.704
P for trend		0.382		0.545
Sb	1.179(0.671-2.029)	0.559	1.186(0.648-2.130)	0.573
Q1				
Q2	1.051(0.565-1.957)	0.875	1.314(0.697-2.504)	0.401
Q3	1.313(0.727-2.396)	0.368	1.534(0.820-2.917)	0.184
Q4	1.414(0.791-2.564)	0.246	1.651(0.877-3.163)	0.123
P for trend		0.180		0.111
Sn	1.231(0.837-1.796)	0.285	1.231(0.810-1.857)	0.325
Q1				
Q2	0.829(0.451-1.512)	0.541	0.772(0.411-1.432)	0.414
Q3	1.088(0.615-1.933)	0.771	1.014(0.559-1.843)	0.962
Q4	1.038(0.584-1.851)	0.898	0.938(0.500-1.753)	0.841
P for trend		0.685		0.944
Tl	1.391(0.731-2.687)	0.320	1.352(0.701-2.646)	0.373
Q1				
Q2	1.098(0.602-2.012)	0.760	1.153(0.624-2.142)	0.649
Q3	1.049(0.571-1.930)	0.877	0.972(0.517-1.824)	0.928
Q4	1.398(0.789-2.508)	0.254	1.468(0.816-2.677)	0.203
P for trend		0.289		0.282
Tu	1.176(0.757-1.813)	0.467	1.265(0.801-1.986)	0.310
Q1				
Q2	0.723(0.393-1.313)	0.290	0.694(0.371-1.277)	0.243
Q3	0.959(0.543-1.692)	0.885	0.986(0.551-1.761)	0.961
Q4	0.954(0.540-1.683)	0.871	0.970(0.536-1.753)	0.920
P for trend		0.901		0.810

Model 1 does not adjust for any factors; Model 2 includes the covariates of age, ethnicity, education level, work situation, ever pregnant, and chronic diseases.

### 3.3 Model variable selection

**Figure 2:**
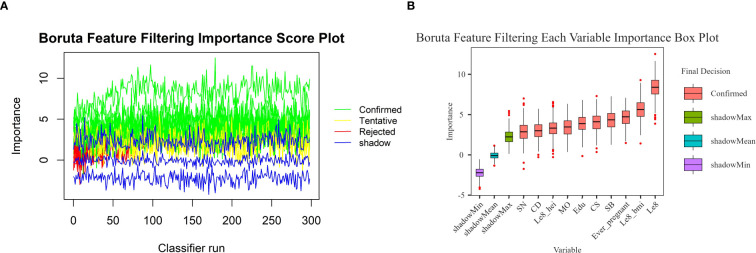
Boruta feature screening plot. **(A)** Importance score plot. **(B)** Importance box plot for each variable.

In this study, the Boruta algorithm with shading was used, the confidence interval was set to 0.01, the source of maximum importance was run 300 times, and the Bonferroni method for multiple comparison adjustment was used to identify 10 potentially effective predictor variables (**Figure 2**). These characteristic variables with shading were used to train and construct the machine learning model, which included education level, pregnancy history, LE8, diet, BMI, Cd, Mo, Sb, Cs and Sn.

### 3.4 Model evaluation and comparison

**Figure 3:**
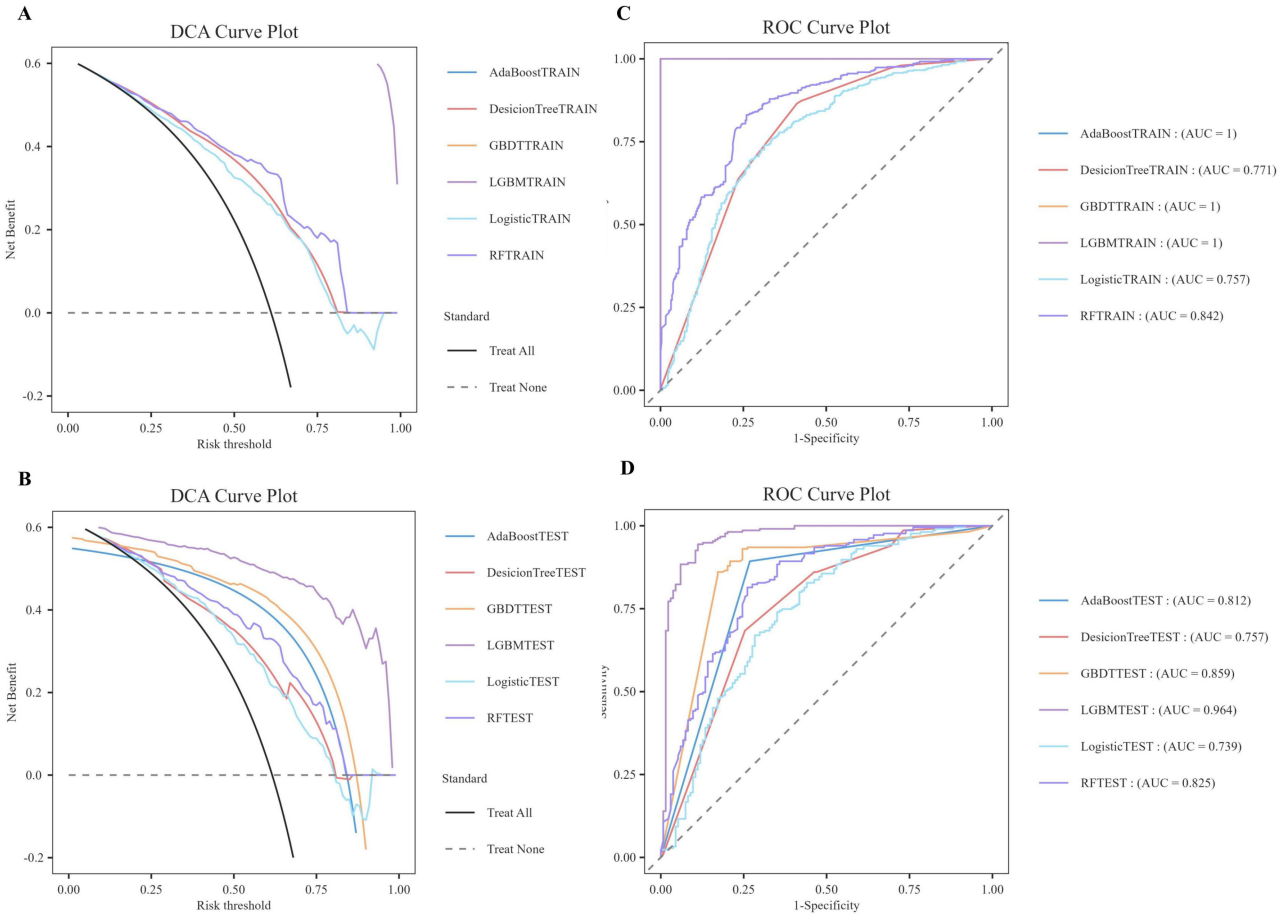
DCA/ROC curves of the train and test sets of 6 ML models. **(A)** DCA curves of the train set. **(B)** DCA curves of the test sets. **(C)** ROC curves of the train set. **(D)** ROC curves of the test sets. A DCA curve that is close to the upper-right corner of the coordinate diagram and a ROC curve that is close to the upper-left corner of the coordinate diagram both indicate good prediction performance.

As illustrated in **Table 6**, the model evaluation results, including Accuracy, Recall, F1 score, and the Matthews Correlation Coefficient (MCC), are presented for both the training set and the test set. **Figures 3A, C** illustrate the DCA curves and ROC curves of the six machine learning models (Decision Tree, GBDT, AdaBoost, LGBM, Logistic Regression, and Random Forest) for the training set, while **Figures 3B, D** depict the DCA curves and ROC curves for the test set. A comprehensive evaluation of the training set and test set using the two curves was conducted, resulting in the identification of satisfactory model evaluation outcomes. LGBM exhibited the optimal prediction performance and pattern.

**Table 6:** Model evaluation for training and test sets.

Model Name	Accuracy	Recall	F1-Score	MCC
Decision Tree TRAIN	0.75739	0.86492	0.81327	0.47744
GBDT TRAIN	1.00000	1.00000	1.00000	1.00000
AdaBoost TRAIN	1.00000	1.00000	1.00000	1.00000
LGBM TRAIN	1.00000	1.00000	1.00000	1.00000
Logistic TRAIN	0.71429	0.84879	0.78399	0.37888
RF TRAIN	0.76970	0.92137	0.83015	0.50680
Decision Tree TEST	0.73639	0.86047	0.80087	0.42537
GBDT TEST	0.84814	0.89302	0.87872	0.67641
AdaBoost TEST	0.83095	0.89302	0.86682	0.63804
LGBM TEST	0.91117	0.96744	0.93065	0.81217
Logistic TEST	0.71920	0.86977	0.79237	0.38346
RF TEST	0.77364	0.93488	0.83576	0.51383

**Figure 4:**
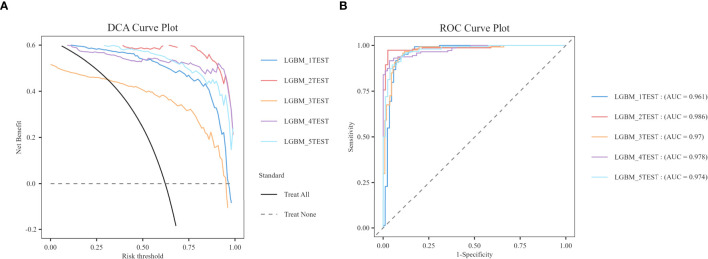
5-fold cross-validation. **(A)** DCA curve. **(B)** ROC curve.

In order to reduce model selection bias and variance and accurately evaluate the performance of machine learning models, the study further utilized 5-fold cross-validation to test the optimal LGBM model. The validation results demonstrate that the LGBM model exhibits excellent performance in prediction, thereby substantiating its high predictive accuracy (**Figure 4**).

### 3.5 Visual analysis of the importance of features

**Figure 5:**
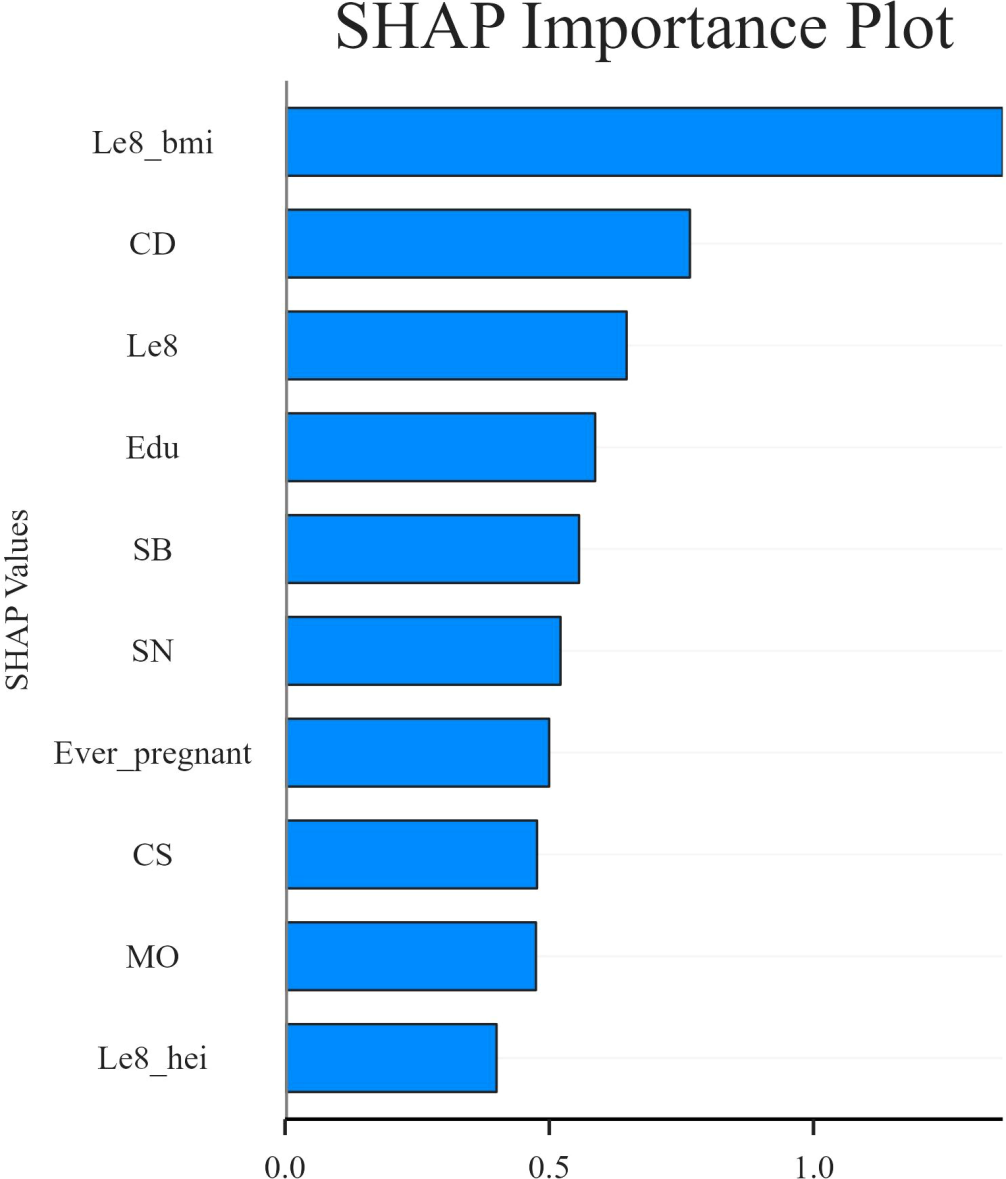
SHAP column chart.

**Figure 6:**
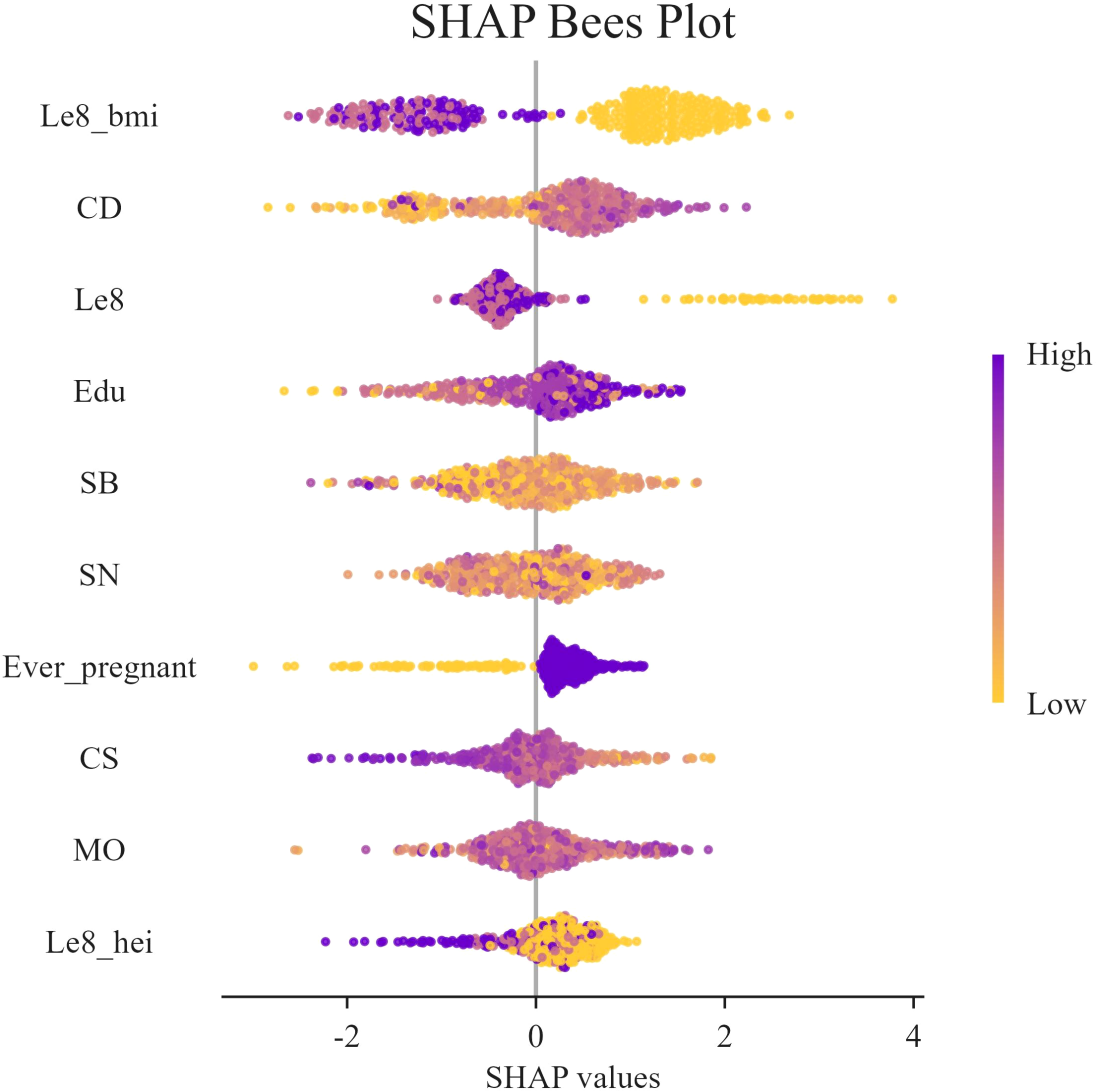
SHAP swarm plot. In the honeycomb diagram, the length of each indicator represents its contribution to infertility prediction, and the color of each point indicates its value magnitude. The 0 coordinate serves as a dividing line; values greater than 0 indicate a positive predictive value, and values less than 0 indicate a negative predictive value.

In this study, SHAP analysis was used to evaluate the contribution and importance of each feature variable in the LGBM model in model prediction. The results are shown in **Figures 5** and **6**. As can be seen in **Figure 5**, BMI, Cd and LE8 have the dominant effect on predicting the risk of female infertility and have the largest SHAP values. The SHAP values are proportional to the effect on the model output, and the importance decreases from top to bottom. In addition, Cs, Mo, Sb, Sn, diet, education level, and pregnancy history also showed significant predictive ability.

**Figure 6** presents the SHAP values distribution of each LE8 indicator, heavy metal exposure, and covariates, as well as their influence on the model output. The findings of the study suggest a negative correlation between BMI, LE8, and Cs with female infertility, while Cd, education level, and pregnancy history exhibit a positive association with infertility. While other variables demonstrate high contribution rates, the mechanisms through which they act remain opaque, and their actions may be related to interactions with other variables.

**Figure 7:**
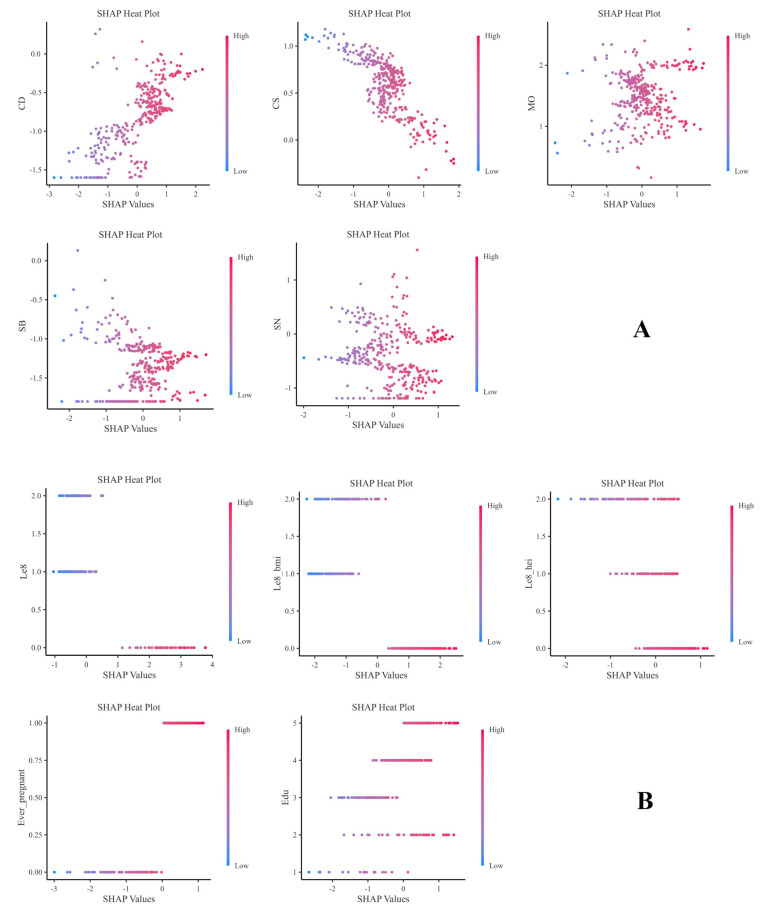
SHAP thermal dispersion diagram **(A, B)**.

**Figure 7** presents the heat maps of each indicator, which clearly show the predictive direction of each factor on infertility. The findings suggest that the predictive directions of each factor align with the results of the honeycomb diagram.

### 3.6 Subgroup analysis of characteristics

**Figure 8:**
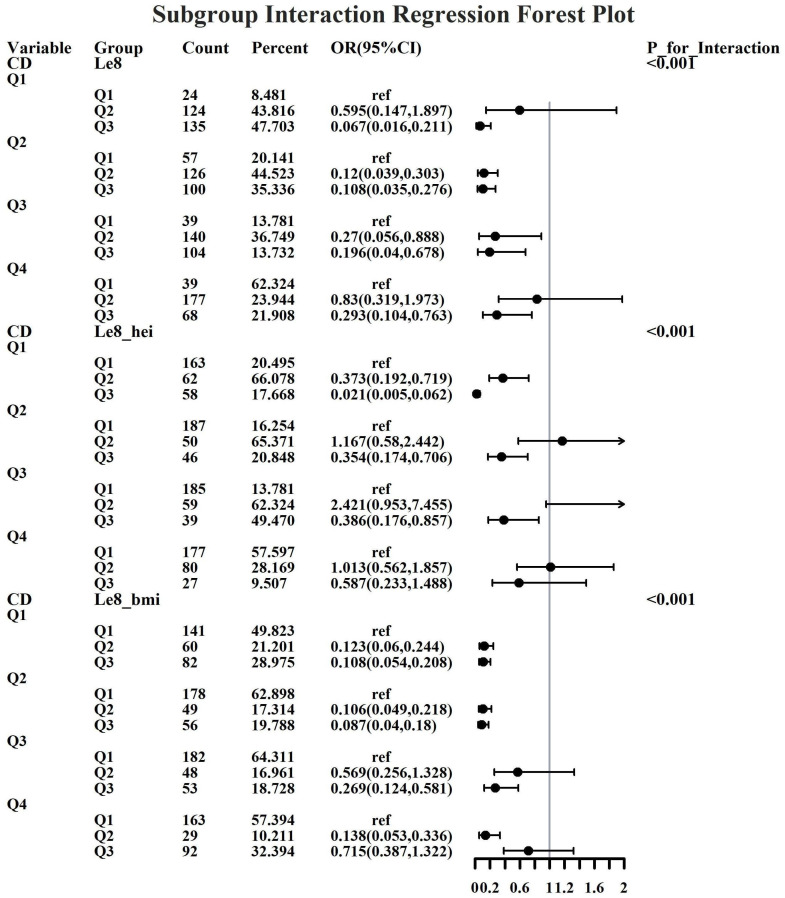
Forest plot of logistic regression based on Cd exposure.

**Figure 9:**
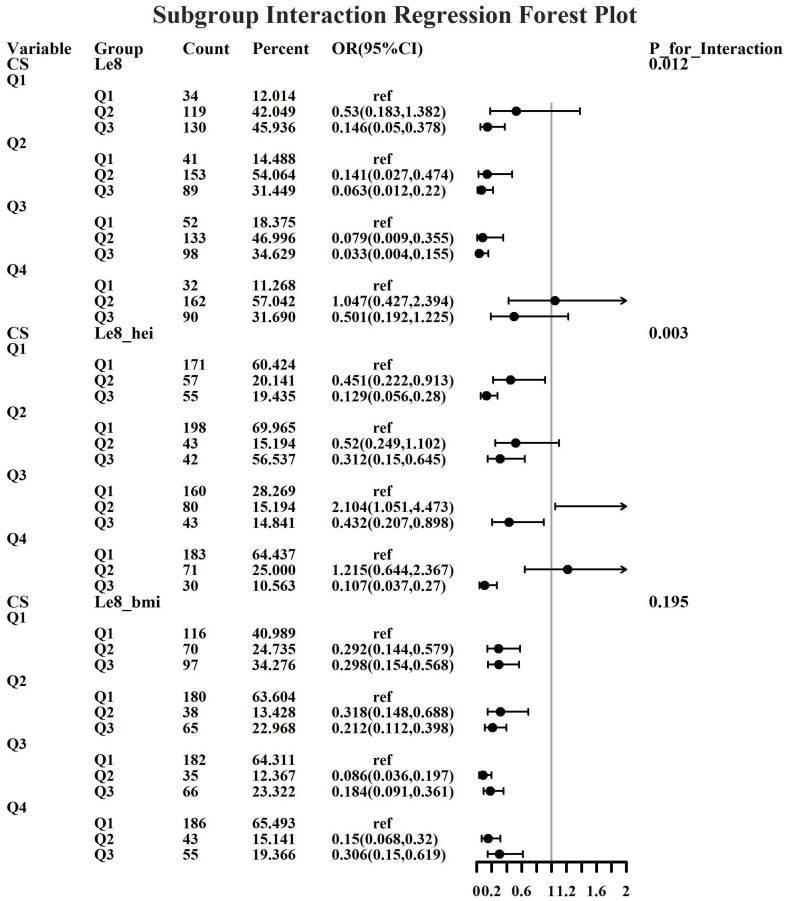
Forest plot of logistic regression based on Cs exposure.

**Figure 10:**
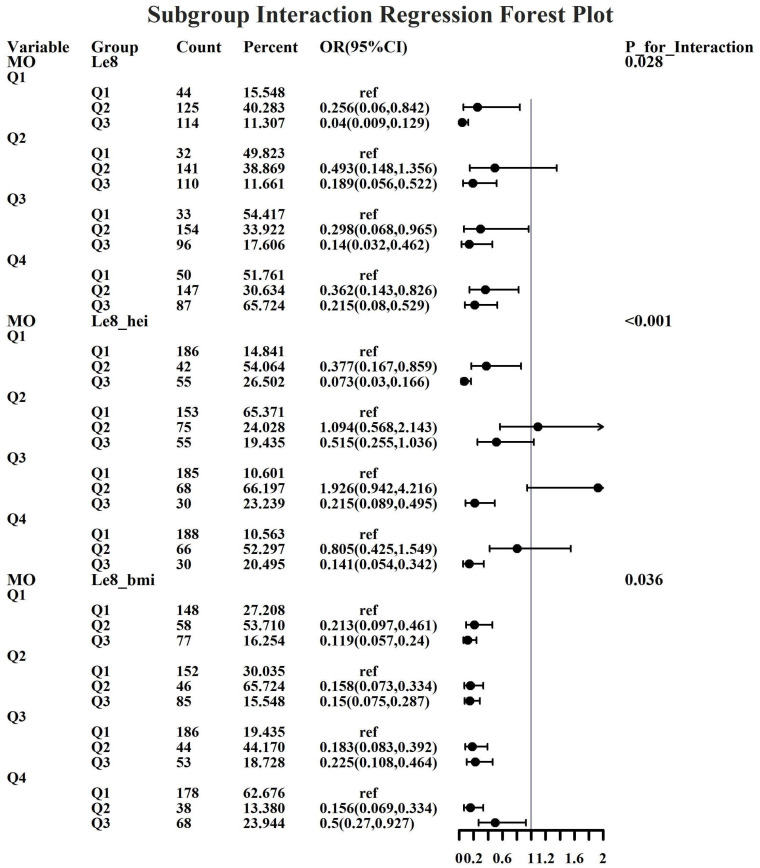
Forest plot of logistic regression based on Mo exposure.

**Figure 11:**
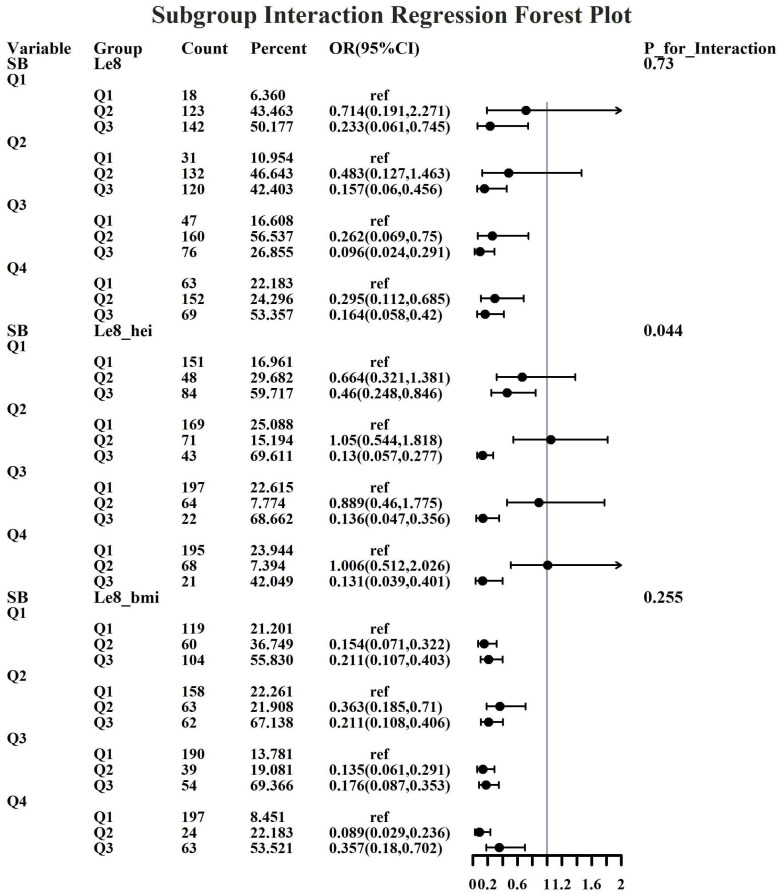
Forest plot of logistic regression based on Sb exposure.

**Figure 12:**
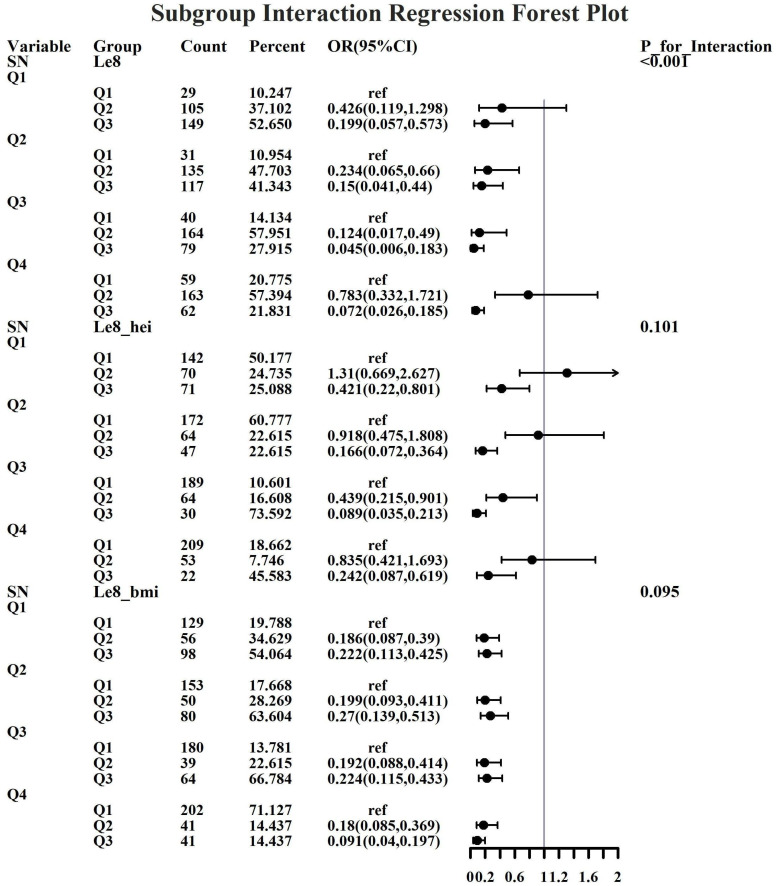
Forest plot of logistic regression based on Sn exposure.

Given the investigation’s objective to assess the joint predictive effect of LE8 and heavy metal exposure on the risk of female infertility, a decision was made to proceed with the execution of subgroup analysis and interaction tests on the identified core predictive variables. The aforementioned core predictive variables consist of the four heavy metal variables (Cd, Mo, Sb, Cs, Sn) and the three LE8 indicators (LE8, diet, BMI). The dataset generated using Synthetic Minority Over-sampling Technique combined with undersampling was utilized for the aforementioned analyses. This investigation was undertaken to ascertain whether LE8 and heavy metal exposure interact to influence female infertility (**Figures 8**–**12**). Preliminary findings indicate that the overall LE8 score demonstrates a substantial degree of interaction with Cd, Cs, Mo, and Sn, as indicated by key indicators in LE8. When the levels of these heavy metals are at the Q1 and Q4 quartiles, LE8 is at a moderate level, which is often associated with infertility risk. However, when LE8 is at a high level, it can reduce infertility risk to some extent. The present study demonstrates that dietary intake exhibits significant interactions with Cd, Cs, Mo, and Sb. When the concentrations of these heavy metals reach the Q2, Q3, and Q4 quartiles, moderate dietary intake is frequently linked to an elevated risk of infertility, while high dietary intake has been observed to reduce the risk of infertility. A significant interaction effect was observed between BMI and Cd. When Cd is at the Q3 percentile, the risk of infertility increases in the group with moderate BMI levels. When Cd is at the Q4 percentile, the risk of infertility increases in the group with high BMI levels.

### 3.7 Sensitivity analysis

It must be acknowledged that extant research findings may be subject to data bias. Furthermore, given that the NHANES database employs self-reported data to measure female infertility symptoms, it is possible that some women may be infertile without realizing it. Therefore, to further validate the robustness of the research findings, propensity score matching (PSM) was used to balance the sample for sensitivity analysis. To ensure a balanced data structure without a significant loss of samples, a 1:8 matching method was employed, with the sample size of the non-infertility group being adjusted to 488. Following a thorough examination of the results, it was determined that the standardized mean differences (SMDs) for the majority of key variables underwent a substantial reduction, falling below 0.25. Furthermore, the intervention and control groups exhibited no substantial disparities following matching, suggesting that the features were well-balanced (**Table 7**).

**Table 7:** Balance test.

Variable name	Matching status	SMD	Intergroup *p*-value
Age	Before matching	0.307	0.003
	After matching	0.166	0.123
Ethnicity	Before matching	0.183	0.573
	After matching	0.120	0.881
Education	Before matching	0.172	0.643
	After matching	0.101	0.933
Work situation	Before matching	0.192	0.825
	After matching	0.063	0.950
Ever pregnant	Before matching	0.347	0.003
	After matching	0.214	0.062
Ba	Before matching	0.116	0.591
	After matching	0.054	0.821
Cd	Before matching	0.212	0.017
	After matching	0.117	0.481
Co	Before matching	0.063	0.433
	After matching	0.053	0.413
Cs	Before matching	0.015	0.804
	After matching	0.011	0.110
Mn	Before matching	0.099	0.505
	After matching	0.063	0.766
Mo	Before matching	0.053	0.942
	After matching	0.058	0.947
Pb	Before matching	0.089	0.828
	After matching	0.055	0.966
Sb	Before matching	0.064	0.484
	After matching	0.045	0.593
Sn	Before matching	0.107	0.719
	After matching	0.050	0.435
Tl	Before matching	0.105	0.505
	After matching	0.073	0.789
Tu	Before matching	0.071	0.696
	After matching	0.035	0.886
Chronic disease	Before matching	0.421	<0.001
	After matching	0.219	0.129
LE8	Before matching	0.313	0.002
	After matching	0.245	0.048
Diet	Before matching	0.097	0.659
	After matching	0.057	0.872
Physical activity	Before matching	0.236	0.179
	After matching	0.124	0.589
Nicotine exposure	Before matching	0.149	0.420
	After matching	0.088	0.730
Sleep health	Before matching	0.270	0.031
	After matching	0.173	0.275
BMI	Before matching	0.476	<0.001
	After matching	0.239	0.098
Blood lipids	Before matching	0.115	0.544
	After matching	0.101	0.645
Blood glucose	Before matching	0.122	0.446
	After matching	0.110	0.552
Blood pressure	Before matching	0.299	0.004
	After matching	0.221	0.088

**Figure 13:**
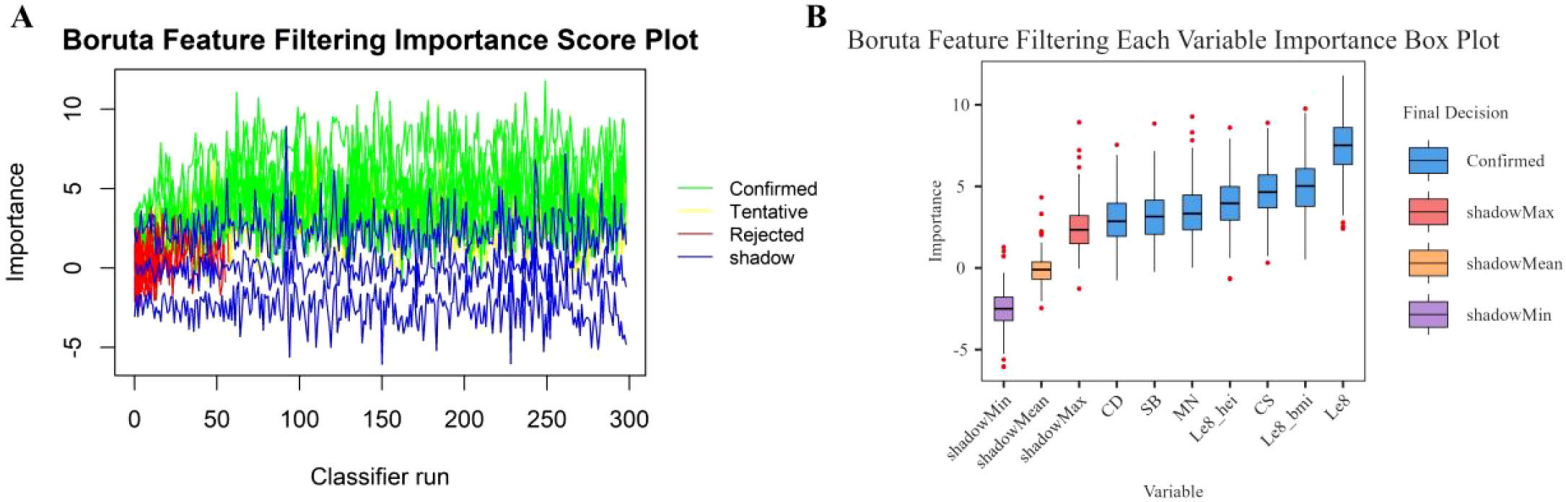
Boruta feature screening plot. **(A)** Importance score plot. **(B)** Importance box plot for each variable.

Subsequent to the initial matching process, the Boruta algorithm was re-implemented, employing the identical parameter settings as previously utilized. The results of the study indicated the presence of LE8, BMI, diet, Cd, Sb, Mn, and Cs. The salient factors identified prior to and following the matching process exhibited substantial consistency, with LE8, BMI, diet, Cd, Sb, and Cs being identified in both instances (**Figure 13**).

**Figure 14:**
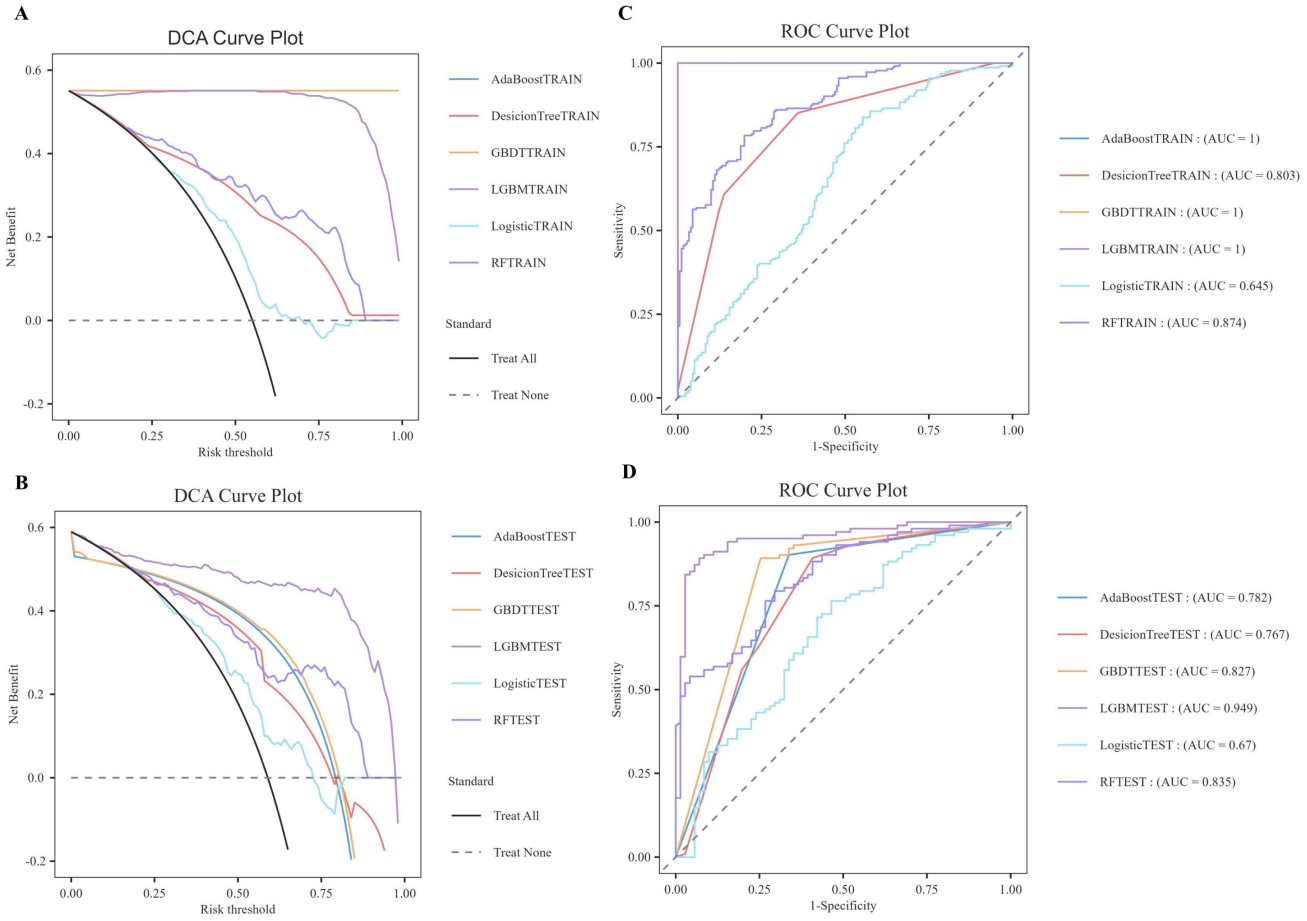
DCA/ROC curves of the train and test sets of 6 ML models. **(A)** DCA curves of the train set. **(B)** DCA curves of the test sets. **(C)** ROC curves of the train set. **(D)** ROC curves of the test sets.

Subsequently, the same six models were used to evaluate the samples. Preliminary findings indicate that, based on the evaluation results, LGBM continues to demonstrate superior predictive capabilities (**Table 8**; **Figure 14**).

**Table 8:** Model evaluation for training and test sets.

Model Name	Accuracy	Recall	F1-Score	MCC
Decision Tree TRAIN	0.75682	0.85135	0.79412	0.50720
GBDT TRAIN	1.00000	1.00000	1.00000	1.00000
AdaBoost TRAIN	1.00000	1.00000	1.00000	1.00000
LGBM TRAIN	1.00000	1.00000	1.00000	1.00000
Logistic TRAIN	0.64764	0.78378	0.71020	0.27869
RF TRAIN	0.77419	0.81532	0.79912	0.54212
Decision Tree TEST	0.76879	0.89216	0.81982	0.51616
GBDT TEST	0.80925	0.90196	0.84793	0.60229
AdaBoost TEST	0.80347	0.90196	0.84404	0.59019
LGBM TEST	0.89017	0.92157	0.90821	0.77210
Logistic TEST	0.65896	0.76471	0.72558	0.28085
RF TEST	0.74566	0.81373	0.79048	0.46886

**Figure 15:**
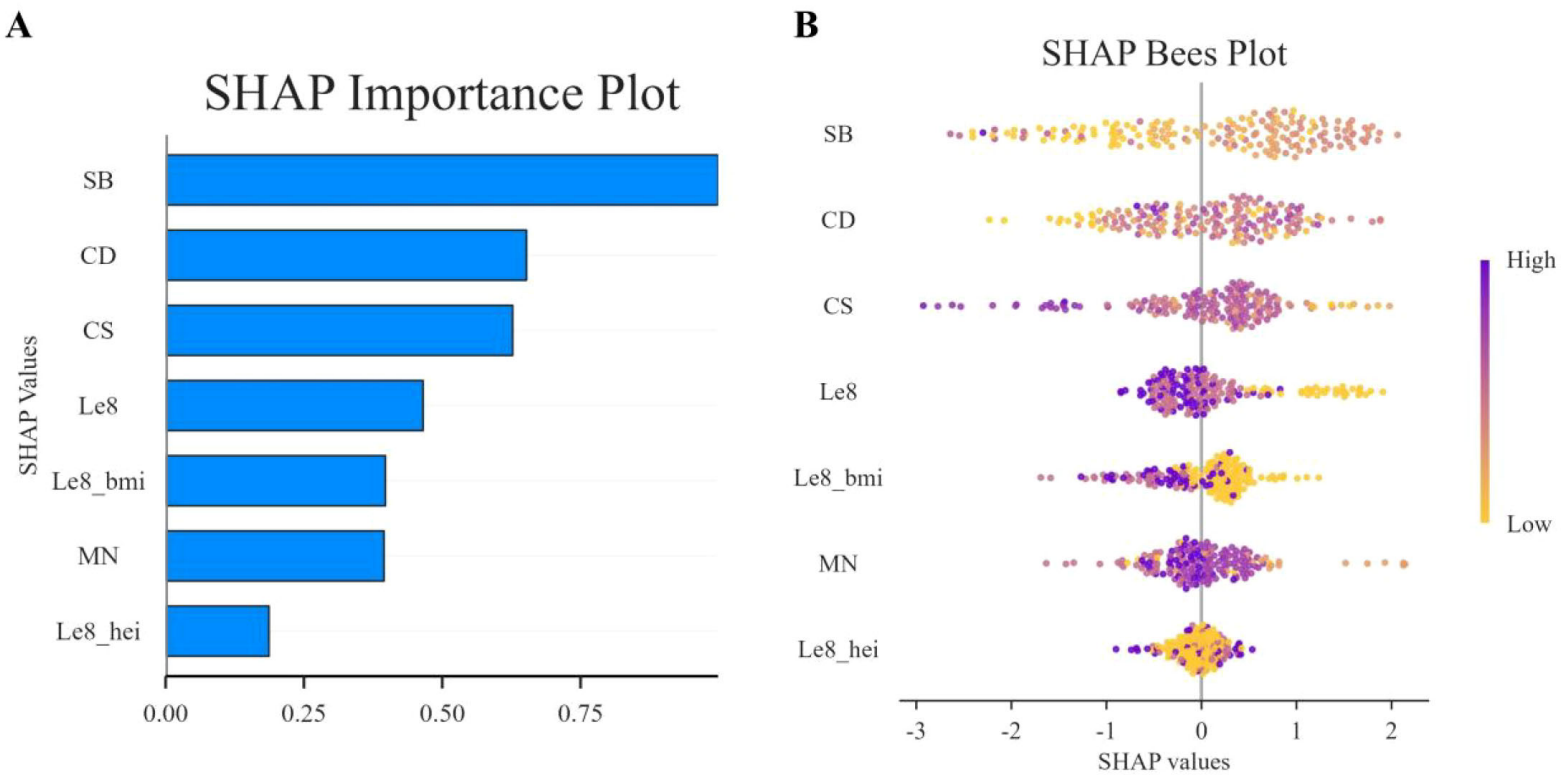
**(A)** SHAP column chart. **(B)** SHAP swarm plot.

Finally, further SHAP explainability analysis was conducted (**Figure 15**), and the results showed that the effects of the six factors (LE8, BMI, diet, Sb, Cd, and Cs) remained consistent with the aforementioned analysis, indicating that the results of this study are robust.

## 4 Discussion

This study utilized data from the US population in the NHANES between 2013 and 2018, in conjunction with the LE8 scoring dimensions, heavy metal exposure, and covariates such as age, education level, work situation, pregnancy history, and chronic disease status, to predict the risk of infertility in U.S. women. The Boruta algorithm was employed to identify ten core variables that significantly contribute to the prediction of infertility: educational level, pregnancy history, LE8 overall, diet, BMI, Cd, Mo, Sb, Cs, and Sn. In light of the aforementioned variables, the training and construction of six machine learning models was undertaken. A comparison of models indicates that the LGBM model exhibits the optimal predictive capacity, suggesting that the LGBM model demonstrates remarkably high precision in forecasting the likelihood of female infertility. In the subsequent SHAP interpretability analysis based on the LGBM model, we found that BMI, LE8, and Cd were the variables that contributed most to the model in terms of LE8 scores and heavy metal exposure, with BMI leading to a higher risk of female infertility. The ensuing discourse will concentrate on the 10 feature variables identified and screened by the Boruta algorithm. It will further argue the potential association between each core variable and infertility risk based on existing epidemiological and toxicological studies.

BMI is a commonly used indicator for assessing an individual’s weight status and degree of obesity, based on the LE8 score. Previous studies have confirmed its close association with female reproductive health, such as ovarian function and the menstrual cycle. Overweight and obese women (BMI≧25kg/m^2^) are more likely to face the risk of infertility, and the risk increases with increasing BMI (23). Obesity has been demonstrated to induce systemic oxidative stress and to precipitate the development of complications through mechanisms that affect the insulin signaling pathway, adipocyte function, and inflammatory response (26). Reproductive organ dysfunction, including decreased ovarian reserve function, diminished egg quality, reduced fertilization capacity, and abnormal embryonic development, has been observed to be associated with obesity (60). Studies have long confirmed that polycystic ovary syndrome is the most common cause of anovulatory infertility in women (23). Obesity exacerbates the metabolic abnormalities of polycystic ovary syndrome, and the prevalence rate among overweight and obese women is as high as 80% (61). Concurrently, obese women demonstrate inferior outcomes when undergoing assisted reproductive technology treatment. Evidence of this includes a diminished response to ovulation-inducing medications, a reduced number of oocytes retrieved, and diminished embryo quality.

Diet is a common indicator used to assess an individual’s health status, nutritional level, and lifestyle characteristics, and is of great importance in research related to women’s reproductive health. Previous studies also support the influence of dietary patterns on women’s reproductive health. On the one hand, a pro-inflammatory diet may affect women’s reproductive health by increasing systemic inflammation (62). For instance, a diet high in sweet beverages, desserts, caffeinated drinks, potatoes, fast food, and other similar items may result in inflammatory responses, obesity, and insulin resistance. Moreover, such dietary habits may increase the risk of fertility in women, particularly in patients diagnosed with polycystic ovary syndrome (63–65). Studies have confirmed that a light fasting diet combined with flaxseed powder supplementation can significantly improve obesity, blood lipid and blood sugar metabolic disorders in patients with polycystic ovary syndrome and combined infertility (66). On the other hand, there is growing evidence that a healthy diet has a positive effect on fertility. A healthy diet, such as the Mediterranean diet, which is rich in whole grains, monounsaturated or polyunsaturated fats, vegetables, fruits, fish, and olive oil, can improve egg quality and quantity through multiple mechanisms, including increasing antioxidant capacity, improving hormone levels, and reducing inflammatory responses, thereby improving fertility success rates (67, 68). At the same time, a healthy diet can help alleviate depressive symptoms, improve physical health, and indirectly improve fertility success rates (69, 70). In addition, the total LE8 score has been validated in multiple studies as a negative predictor of female infertility. There is a significant negative correlation between overall LE8 score and female infertility. Women with better cardiovascular health are 41% less likely to suffer from infertility than those with poorer cardiovascular health (22, 71), Infertility at a young age may have a greater impact on long-term cardiovascular health (21). As the LE8 score increases, there is a concomitant improvement in the cardiovascular health of the woman, as well as an enhancement in her overall lifestyle, both of which have a beneficial effect on her reproductive health. The synergistic effect of multiple factors has been demonstrated to engender improvements in fertility and to reduce the risk of infertility.

In this model, we found an association between Cd, Mo, Sb, Cs and Sn exposure and the risk of female infertility. Cd showed a positive contribution to the prediction of the risk of female infertility. Previous research has provided the first evidence of a positive association between cadmium exposure and the risk of primary ovarian insufficiency in the Chinese population (72). Cd exposure not only induces oxidative stress in the reproductive organs, but also interferes with the synthesis and secretion of reproductive hormones, thereby affecting reproductive function (73–75). Some research even shows that even low levels of cadmium in the blood can significantly affect women’s reproductive health and increase the risk of infertility (76). The findings of this study offer convergent evidence in support of previous findings and extend them by providing an exotic validation. That is to say, they demonstrate a positive association between cadmium exposure and the risk of female infertility in a U.S. population.

Mo, Sb and Sn demonstrate a relatively intricate nonlinear relationship, with both exhibiting a two-way risk prediction for the risk of female infertility. Existing studies have shown a non-linear relationship between Mo concentration and infertility. Previous studies have shown that urinary molybdenum concentrations significantly affect clinical *in vitro* fertilization outcomes. Higher concentrations of molybdenum are associated with lower implantation rates, clinical pregnancy rates, and live birth rates (77). Later studies found that the concentration of trace elements such as Mo in the urine of women with premature ovarian failure was significantly lower than that of healthy controls, and that these elements may be related to the development of premature ovarian failure (78). The two studies show very different results. A further interpretation of the results shows that the excess Mo in the first study had a toxic effect on the reproductive system, affecting the ovarian response and embryonic development, and thus the outcome of assisted reproduction. Mo in the latter study is one of the essential trace elements in the human body, involved in the activity of various enzymes and metabolic processes. If Mo levels are low, it can negatively affect fertility by interfering with the activity of key enzymes. Therefore, the association between Mo and infertility needs to be further explored and verified with larger sample sizes and more mechanistic studies. Previous studies have suggested that Sb exposure may contribute to the development of polycystic ovary syndrome by inducing oxidative stress and disrupting redox homeostasis, thereby increasing the risk of infertility (79). Subsequent studies have also shown a significant non-linear relationship (*p*<0.05) between Ba, Mo, and Sb and infertility (80). However, no studies have systematically investigated the relationship between Sb content and the risk of infertility. Research has demonstrated that tin accumulation in the human body may have deleterious effects on reproductive health, given its classification as a non-essential trace element. Epidemiological studies have demonstrated that women with long-term exposure to or intake of higher doses of tin exhibit significantly increased tin concentrations in urine. This is closely associated with reduced ovarian response, diminished embryonic development potential, and lower live birth rates (81). Additionally, Chi et al. (2025) utilized NHANES database data to further discover that for every increase of one quartile range in urinary tin concentration, the risk of female infertility correspondingly increased by 8% (80).

Finally, although the mechanism between Cs and female infertility is not yet clear, the SHAP heat map (**Figure 7**) of this study clearly shows that Cs content in the fourth quartile has a negative correlation with female infertility. However, a subsequent analysis of subgroups and interaction testing (**Figure 9**) revealed that within the fourth quartile, where LE8 exhibited a moderate score indicative of a general state of health, an elevated Cs content was found to be associated with a heightened risk of infertility. This finding suggests that the effect of Cs on female fertility may not be a simple linear relationship, but may be modulated by an individual’s overall health status or other unknown factors. Therefore, future research needs to further explore the complex relationship between Cs and female infertility and whether this relationship is disrupted or influenced by other factors. In addition, when assessing reproductive health risks, it is important to consider the effects of individual and combined exposures to multiple heavy metals and their specific mechanisms of action under different conditions.

Educational level and pregnancy history are important covariates for predicting the risk of female infertility. However, the current research results show that both are confounding factors. Combined with previous research, education level may be negatively correlated with the risk of female infertility. A correlation has been observed between a higher level of education and a lower risk of infertility in women. This phenomenon may be attributed to the fact that women with a higher level of education have a deeper understanding of health issues and are more inclined to seek medical help (82). A correlation has been demonstrated between a lower level of education and an increased prevalence of female sexual dysfunction, which can impact fertility. This phenomenon may be attributed, at least in part, to a deficiency in knowledge regarding sexual health. The enhancement of educational initiatives and the dissemination of information pertaining to sexual health may prove effective in the amelioration of sexual dysfunction (83). A history of pregnancy may be positively associated with the risk of female infertility. A number of studies have indicated that patients with a medical history of ectopic pregnancy are 62% more likely to experience implantation window displacement compared to those without such a history. This heightened risk can be attributed to the fact that implantation window displacement can impede the successful implantation of the embryo in the endometrium at the optimal time, consequently resulting in implantation failure and subsequent infertility (84). In addition, a history of ectopic pregnancy, polycystic ovary syndrome, and primary infertility are important risk factors for secondary infertility. However, further research is necessary to elucidate the intricate relationship and specific mechanisms between these covariates and the risk of infertility. Such studies are crucial for comprehending the multifaceted influences on female infertility and for developing effective clinical prediction and prevention strategies.

This study employed data from the NHANES to investigate the association between LE8 and heavy metal exposure and female infertility, with a sample that has clear regional representativeness. Despite the absence of specific information on the causes of infertility, the study population includes women aged 20–45 years, thereby enabling the generalizability of the findings to U.S. women of a similar age group. While dietary patterns, BMI, and heavy metal exposure vary across countries, these factors are globally prevalent, and their associations with female reproductive health have been validated in multiple countries and ethnic groups, suggesting a degree of cross-cultural applicability. Meanwhile, the female infertility risk prediction model developed by this institute utilizes publicly available and easily accessible NHANES data, offering an economical and efficient solution for the initial screening and early risk warning of female infertility, thereby realizing a health management model of “proactive identification and precise intervention”. Additionally, the controllable factors identified by the model, such as BMI, dietary habits, and heavy metal exposure, provide data support and guidance for policy-making and personalized reproductive health education. Consequently, these findings provide novel insights that could inform future public health initiatives aimed at the early detection and prevention of female infertility.

## 5 Conclusion

In our study, we analyzed data from the NHANES database, combined the LE8 score and heavy metal exposure indicators, and used a stepwise machine learning strategy to construct a joint prediction model for the risk of female infertility. The conclusions of this study must be interpreted in light of its limitations. Firstly, the NHANES database is characterized by a retrospective and observational design, which precludes the capacity to observe temporal changes in variables. Further prospective validation and supplementation are necessary to enhance the robustness and comprehensiveness of the developed model. Secondly, the aetiology of female infertility is predicated on self-reported data, and the underlying causes remain to be elucidated. Despite the utilization of sensitivity analysis in the study to evaluate the model’s performance, additional external validation is necessary to ensure its robustness and reliability. Moreover, the NHANES database does not currently encompass specific surveys concerning women’s exposure to heavy metals. Subsequent analyses could be conducted by combining hospital data. While the LGBM model has been shown to demonstrate efficacy in predicting infertility risk using NHANES data, its effectiveness in other datasets or application scenarios remains to be fully validated. In subsequent research, we anticipate implementing model transfer learning and external validation based on multi-country datasets to enhance the model’s generalization capability and prediction accuracy. Concurrently, we will enhance the application of various machine learning technologies to improve the interpretability and robustness of the model, ensuring its reliability in diverse datasets and application scenarios.

## Data Availability

The datasets presented in this study can be found in online repositories. The names of the repository/repositories and accession number(s) can be found in the article/Supplementary Material.

## Appendix

**Table:**

Abbreviations	Designation
LE8	Life’s essential 8
AdaBoost	Adaptive Boosting
GBDT	Gradient Boosting Decision Tree
LGBM	Light Gradient Boosting Machine
AUC	Area Under the Receiver Operating Characteristic Curve
MCC	Matthews Correlation Coefficient
SHAP	SHapley Additive exPlanations
ML	Machine Learning
BMI	Body Mass Index
NHANES	National Health and Nutrition Examination Survey

**Table:**

Indicators description	Designation
Le8 bmi	Body Mass Index, BMI
Le8 pa	Physical Activity
Le8 hei	Diet
Le8 sleep	Sleep Health
Le8 smoke	Nicotine Exposure
Le8 bp	Blood Pressure
Le8 glucose	Blood Glucose
Le8 non_hdl	Blood Lipids(Non-High-Density Lipoproteinemia)
BA	Barium
CD	Cadmium
CO	Cobalt
CS	Cesium
MN	Manganese
MO	Molybdenum
PB	Lead
SB	Antimony
SN	Tin
TL	Thallium
TU	Titanium

## References

[1] Krueger RB Reed GM First MB Marais A Kismodi E Briken P. Proposals for paraphilic disorders in the international classification of diseases and related health problems, eleventh revision (ICD-11). Arch Sexual Behav. (2017) 46:1529–45. doi: 10.1007/s10508-017-0944-2 PMC5487931 28210933

[2] Shen DY Yang S Qi C. Global, regional, and national prevalence and disability-adjusted life-years for female infertility: Results from a global burden of disease study, 1990-2019. Gynecol Obstetr Invest. (2024), 1–21. doi: 10.1159/000542408 PMC12324765 39571567

[3] Carson SA Kallen AN. Diagnosis and management of infertility: a review. Jama. (2021) 326:65–76. doi: 10.1001/jama.2021.4788 34228062 PMC9302705

[4] Zhao YX Chen SR Su PP Huang FH Shi YC Shi QY. Using mesenchymal stem cells to treat female infertility: an update on female reproductive diseases. Stem Cells Int. (2019) 1:9071720. doi: 10.1155/2019/9071720 PMC6925937 31885630

[5] Rezvani M Shaaban AM. Fallopian tube disease in the nonpregnant patient. Radiographics. (2011) 31:527–48. doi: 10.1148/rg.312105090 21415195

[6] Tarín JJ García-Pérez MA Hamatani T Cano A. Infertility etiologies are genetically and clinically linked with other diseases in single meta-diseases. Reprod Biol Endocrinol. (2015) 13:1–11. doi: 10.1186/s12958-015-0029-9 25880215 PMC4404574

[7] Mascarenhas MN Flaxman SR Boerma T Vanderpoel S Stevens GA. National, regional, and global trends in infertility prevalence since 1990: a systematic analysis of 277 health surveys. PloS Med. (2012) 9:e1001356. doi: 10.1371/journal.pmed.1001356 23271957 PMC3525527

[8] Woodruff TJ Zota AR Schwartz JM. Environmental chemicals in pregnant women in the United States: NHANES 2003-2004. Environ Health Perspect. (2011) 119:878–85. doi: 10.1289/ehp.1002727 PMC3114826 21233055

[9] Teklemicheal AG Kassa EM Weldetensaye EK. Prevalence and correlates of infertility related psychological stress in women with infertility: a cross-sectional hospital based survey. BMC Psychol. (2022) 10:91. doi: 10.1186/s40359-022-00804-w 35392978 PMC8988399

[10] Liat LG Jaron R Liraz O Tzvia B Shlomo M Bruno L. Are infertility treatments a potential risk factor for cancer development? Perspective of 30 years of follow-up. Gynecol Endocrinol. (2012) 28:809–14. doi: 10.3109/09513590.2012.671391 22475084

[11] dos Santos Silva I Wark PA McCormack VA Mayer D Overton C Little V. Ovulation-stimulation drugs and cancer risks: a long-term follow-up of a British cohort. Br J Cancer. (2009) 100:1824–31. doi: 10.1038/sj.bjc.6605086 PMC2695698 19436296

[12] Althuis MD Moghissi KS Westhoff CL Scoccia B Lamb EJ Lubin JH. Uterine cancer after use of clomiphene citrate to induce ovulation. Am J Epidemiol. (2005) 161:607–15. doi: 10.1093/aje/kwi084 15781949

[13] Lloyd-Jones DM Allen NB Anderson CAM Black T Brewer LC Foraker RE. Life’s essential 8: updating and enhancing the American Heart Association’s construct of cardiovascular health: a presidential advisory from the American Heart Association. Circulation. (2022) 146:e18–43. doi: 10.1161/CIR.0000000000001078 PMC10503546 35766027

[14] Sun J Li Y Zhao M Yu X Zhang C Magnussen CG. Association of the American Heart Association’s new “Life’s Essential 8” with all-cause and cardiovascular disease-specific mortality: prospective cohort study. BMC Med. (2023) 21:116. doi: 10.1186/s12916-023-02824-8 36978123 PMC10053736

[15] Isiozor NM Kunutsor SK Voutilainen A Laukkanen JA Notes A. Life’s Essential 8 and the risk of cardiovascular disease death and all-cause mortality in Finnish men. Eur J Prev Cardiol. (2023) 30:658–67. doi: 10.1093/eurjpc/zwad040 36753230

[16] Yi J Wang L Guo X Ren XP. Association of Life’s Essential 8 with all-cause and cardiovascular mortality among US adults: A prospective cohort study from the NHANES 2005-2014. Nutrition Metab Cardiovasc Dis. (2023) 33:1134–43. doi: 10.1016/j.numecd.2023.01.021 36775706

[17] Ma H Wang X Xue Q Li X Liang Z Heianza Y. Cardiovascular health and life expectancy among adults in the United States. Circulation. (2023) 147:1137–46. doi: 10.1161/CIRCULATIONAHA.122.062457 PMC10165723 37036905

[18] Wang X Ma H Li X Heianza Y Manson JE Franco OH. Association of cardiovascular health with life expectancy free of cardiovascular disease, diabetes, cancer, and dementia in UK adults. JAMA Internal Med. (2023) 183:340–9. doi: 10.1001/jamainternmed.2023.0015 PMC9972243 36848126

[19] Petermann-Rocha F Deo S Celis-Morales C Ho FK Bahuguna P McAllister D. An opportunity for prevention: associations between the Life’s Essential 8 score and cardiovascular incidence using prospective data from UK Biobank. Curr Problems Cardiol. (2023) 48:101540. doi: 10.1016/j.cpcardiol.2022.101540 36528209

[20] Jennifer L Beckie TM Brown HL Brown SD Garovic VD Khan SS. Opportunities in the postpartum period to reduce cardiovascular disease risk after adverse pregnancy outcomes: a scientific statement from the American Heart Association. Circulation. (2024) 149:e330–46. doi: 10.1161/CIR.0000000000001212 PMC11185178 38346104

[21] Nichols AR Rifas-Shiman SL Switkowski KM Zhang MY Young JG Hivert MF. History of infertility and midlife cardiovascular health in female individuals. JAMA Netw Open. (2024) 7:e2350424–e2350424. doi: 10.1001/jamanetworkopen.2023.50424 38180761 PMC10770770

[22] Luo M Li JS Xiao XJ Wu P Zhang Y. Associations between cardiovascular health and female infertility: A national population-based study. PloS One. (2024) 19:e0306476. doi: 10.1371/journal.pone.0306476 38968246 PMC11226045

[23] Balen AH Morley LC Misso M Franks S Legro RS Wijeyaratne CN. The management of anovulatory infertility in women with polycystic ovary syndrome: an analysis of the evidence to support the development of global WHO guidance. Hum Reprod Update. (2016) 22:687–708. doi: 10.1093/humupd/dmw025 27511809

[24] Langley-Evans SC Pearce J Ellis S. Overweight, obesity and excessive weight gain in pregnancy as risk factors for adverse pregnancy outcomes: A narrative review. J Hum Nutr Dietetics. (2022) 35:250–64. doi: 10.1111/jhn.12999 PMC9311414 35239212

[25] Broughton DE Moley KH. Obesity and female infertility: potential mediators of obesity’s impact. Fertil Steril. (2017) 107:840–7. doi: 10.1016/j.fertnstert.2017.01.017 28292619

[26] Arias A Quiroz A Santander N Morselli E Busso D. Implications of high-density cholesterol metabolism for oocyte biology and female fertility. Front Cell Dev Biol. (2022) 10:941539. doi: 10.3389/fcell.2022.941539 36187480 PMC9518216

[27] Butt MS Saleem J Zakar R Aiman S Khan MZ Fischer F. Benefits of physical activity on reproductive health functions among polycystic ovarian syndrome women: a systematic review. BMC Public Health. (2023) 23:882. doi: 10.1186/s12889-023-15730-8 37173672 PMC10176874

[28] Ryan DH Yockey SR. Weight loss and improvement in comorbidity: differences at 5%, 10%, 15%, and over. Curr Obes Rep. (2017) 6:187–94. doi: 10.1007/s13679-017-0262-y PMC5497590 28455679

[29] Singh S Pal N Shubham S Sarma D Verma V Marotta F. Polycystic ovary syndrome: etiology, current management, and future therapeutics. J Clin Med. (2023) 12:1454. doi: 10.3390/jcm12041454 36835989 PMC9964744

[30] Li J Huang Y Xu SR Wang Y. Sleep disturbances and female infertility: a systematic review. BMC Women’s Health. (2024) 24:643. doi: 10.1186/s12905-024-03508-y 39707272 PMC11660991

[31] He S Wan L. Associations between smoking status and infertility: a cross-sectional analysis among USA women aged 18–45 years. Front Endocrinol. (2023) 14:1140739. doi: 10.3389/fendo.2023.1140739 PMC10168125 37181041

[32] Soria-Contreras DC Oken E Tellez-Rojo MM Rifas-Shiman SL Perng W Chavarro JE. History of infertility and long-term weight, body composition, and blood pressure among women in Project Viva. Ann Epidemiol. (2022) 74:43–50. doi: 10.1016/j.annepidem.2022.06.033 35777630 PMC9509485

[33] Järup L. Hazards of heavy metal contamination. Br Med Bull. (2003) 68:167–82. doi: 10.1093/bmb/ldg032 14757716

[34] Kim J-J Kim Y-S Kumar V. Heavy metal toxicity: An update of chelating therapeutic strategies. J Trace Elements Med Biol. (2019) 54:226–31. doi: 10.1016/j.jtemb.2019.05.003 31109617

[35] Paithankar JG Saini S Dwivedi S Sharma A Chowdhuri DK. Heavy metal associated health hazards: An interplay of oxidative stress and signal transduction. Chemosphere. (2021) 262:128350. doi: 10.1016/j.chemosphere.2020.128350 33182141

[36] Dong Q Fu H Jiang H. The role of exosome-shuttled miRNAs in heavy metal-induced peripheral tissues and neuroinflammation in Alzheimer’s disease. Biomed Pharmacother. (2024) 176:116880. doi: 10.1016/j.biopha.2024.116880 38850652

[37] Pan Z Gong T Liang P. Heavy metal exposure and cardiovascular disease. Circ Res. (2024) 134:1160–78. doi: 10.1161/CIRCRESAHA.123.323617 38662861

[38] Zheng KY Zeng ZJ Tian QW Huang JT Zhong Q Huo X. Epidemiological evidence for the effect of environmental heavy metal exposure on the immune system in children. Sci Total Environ. (2023) 868:161691. doi: 10.1016/j.scitotenv.2023.161691 36669659

[39] Planchart A Green A Hoyo C Mattinglyet CJ. Heavy metal exposure and metabolic syndrome: evidence from human and model system studies. Curr Environ Health Rep. (2018) 5:110–24. doi: 10.1007/s40572-018-0182-3 PMC6053628 29460222

[40] Sulagna D Gorain B Choudhury H Roychoudhury S Sengupta P. Environmental and occupational exposure of metals and female reproductive health. Environ Sci pollut Res. (2022) 29:62067–92. doi: 10.1007/s11356-021-16581-9 34558053

[41] Kirmizi DA Baser E Turksoy VA Kara M Yalvac ES Gocmen AY. Are heavy metal exposure and trace element levels related to metabolic and endocrine problems in polycystic ovary syndrome? Biol Trace Element Res. (2020) 198:77–86. doi: 10.1007/s12011-020-02220-w 32504400

[42] Canipari R De Santis L Cecconi S. Female fertility and environmental pollution. Int J Environ Res Public Health. (2020) 17:8802. doi: 10.3390/ijerph17238802 33256215 PMC7730072

[43] Hong X Wang W Huang LL Yuan JH Ding XL Wang H. Associations between multiple metal exposure and fertility in women: A nested case-control study. Ecotoxicol Environ Saf. (2024) 272:116030. doi: 10.1016/j.ecoenv.2024.116030 38310826

[44] Zhang Y Li S Li S. Relationship between cadmium content in semen and male infertility: a meta-analysis. Environ Sci pollut Res. (2019) 26:1947–53. doi: 10.1007/s11356-018-3748-6 30460654

[45] Jain RB. Effect of pregnancy on the levels of blood cadmium, lead, and mercury for females aged 17–39 years old: data from National Health and Nutrition Examination Survey 2003-2010. J Toxicol Environ Health Part A. (2013) 76:58–69. doi: 10.1080/15287394.2012.722524 23151210

[46] Lin J Lin XY Qiu JH You XM Xu JB. Association between heavy metals exposure and infertility among American women aged 20–44 years: A cross-sectional analysis from 2013 to 2018 NHANES data. Front Public Health. (2023) 11:1122183. doi: 10.3389/fpubh.2023.1122183 36866101 PMC9971928

[47] Chen YM Xu C Huang Y Liu ZY Zou JP Zhu HL. The adverse impact of bisphenol A exposure on optimal cardiovascular health as measured by life’s essential 8 in US adults: evidence from NHANES 2005 to 2016. Nutrients. (2024) 16:3253. doi: 10.3390/nu16193253 39408220 PMC11478777

[48] Aimuzi R Xie ZL Qu YM Jiang Y. Air pollution, life’s essential 8, and risk of severe non-alcoholic fatty liver disease among individuals with type 2 diabetes. BMC Public Health. (2024) 24:1350. doi: 10.1186/s12889-024-18641-4 38769477 PMC11103844

[49] Xue TT Wang LM Zhang X Zhao ZP Qi JL Li C. Ambient fine particulate matter and Life’s essential 8 and mortality in adults in China: A Nationwide retrospective cohort study. Prev Med. (2024) 186:108094. doi: 10.1016/j.ypmed.2024.108094 39122017

[50] Trigka M Dritsas E. Long-term coronary artery disease risk prediction with machine learning models. Sensors. (2023) 23:1193. doi: 10.3390/s23031193 36772237 PMC9920214

[51] Shu L Yan H Wu YZ Yan TF Yang L Zhang S. Explainable machine learning in outcome prediction of high-grade aneurysmal subarachnoid hemorrhage. Aging (Albany NY). (2024) 16:4654. doi: 10.18632/aging.205621 38431285 PMC10968679

[52] Li X Zhao Y Zhang DD Kuang L Huang H Chen WL. Development of an interpretable machine learning model associated with heavy metals’exposure to identify coronary heart disease among US adults via SHAP: Findings of the US NHANES from 2003 to 2018. Chemosphere. (2023) 311:137039. doi: 10.1016/j.chemosphere.2022.137039 36342026

[53] Ejiyi CJoseph Qin Z Ukwuoma CC Nneji GU Monday HN Ejiyic MB. Comparative performance analysis of Boruta, SHAP, and Borutashap for disease diagnosis: a study with multiple machine learning algorithms. Netw: Comput Neural Syst. (2024), 1–38. doi: 10.1080/0954898X.2024.2331506 38511557

[54] Ahluwalia N Dwyer J Terry A Moshfegh A Johnson C. Update on NHANES dietary data: focus on collection, release, analytical considerations, and uses to inform public policy. Adv Nutr. (2016) 7:121–34. doi: 10.3945/an.115.009258 PMC4717880 26773020

[55] Keil AP Buckley JP O’Brien KM Ferguson KK Zhao SS White AJ. A quantile-based g-computation approach to addressing the effects of exposure mixtures. Environ Health Perspect. (2020) 128:4. doi: 10.1289/EHP5838 PMC7228100 32255670

[56] Vrigazova B. The proportion for splitting data into training and test set for the bootstrap in classification problems. Business Syst Res: Int J Soc Adv Innovation Res Economy. (2021) 12:228–42. doi: 10.2478/bsrj-2021-0015

[57] Speiser JL Miller ME Tooze J Ip E. A comparison of random forest variable selection methods for classification prediction modeling. Expert Syst Appl. (2019) 134:93–101. doi: 10.1016/j.eswa.2019.05.028 32968335 PMC7508310

[58] Pruessner JC Kirschbaum C Meinlschmid G Hellhammer DH. Two formulas for computation of the area under the curve represent measures of total hormone concentration versus time-dependent change. Psychoneuroendocrinology. (2003) 28:916–31. doi: 10.1016/S0306-4530(02)00108-7 12892658

[59] Jung Y Hu J. AK-fold averaging cross-validation procedure. J Nonparametric Stat. (2015) 27:167–79. doi: 10.1080/10485252.2015.1010532 PMC5019184 27630515

[60] Silvestris E de Pergola G Rosania R Loverro G. Obesity as disruptor of the female fertility. Reprod Biol Endocrinol. (2018) 16:1–13. doi: 10.1186/s12958-018-0336-z 29523133 PMC5845358

[61] Osibogun O Ogunmoroti O Michos ED. Polycystic ovary syndrome and cardiometabolic risk: Opportunities for cardiovascular disease prevention. Trends Cardiovasc Med. (2020) 30:399–404. doi: 10.1016/j.tcm.2019.08.010 31519403

[62] Moludi J Kamari N Darbandi M Mostafaei S Moradi S Pasdar Y. Association between dietary inflammatory index and infertility of women; Results from RaNCD Cohort Study. Nutr J. (2023) 22:35. doi: 10.1186/s12937-023-00865-6 37481550 PMC10362741

[63] Jahangirifar M Taebi M Nasr-Esfahani MH Askari G. Dietary patterns and the outcomes of assisted reproductive techniques in women with primary infertility: a prospective cohort study. Int J Fertil Steril. (2018) 12:316. doi: 10.22074/ijfs.2019.5373 30291693 PMC6186288

[64] Lu JY Tang JT Zou YT Wu RC Chen H Wang WJ. Association between dietary inflammatory index and self-reported female infertility from the National Health and Nutrition Examination Survey 2013-2020. J Hum Nutr Dietetics. (2024) 37:354–64. doi: 10.1111/jhn.13261 37897115

[65] Martina C Zambella E Fietta I Inversetti A Simone ND. Dietary patterns and fertility. Biology. (2024) 13:131. doi: 10.3390/biology13020131 38392349 PMC10886842

[66] Jiang XM Wang YF Tang H Ma J Li HY. Effect of light fasting diet therapy on lipid metabolism and sex hormone levels in patients with polycystic ovary syndrome combined with infertility. Gynecol Endocrinol. (2025) 41:2458084. doi: 10.1080/09513590.2025.2458084 39878338

[67] Salvaleda-Mateu M Rodríguez-Varela C Labarta E. Do popular diets impact fertility? Nutrients. (2024) 16:1726. doi: 10.3390/nu16111726 38892663 PMC11174414

[68] Skowrońska M Pawłowski M Milewski R. A literature review and a proposed classification of the relationships between ovulatory infertility and lifestyle factors based on the three groups of ovulation disorders classified by WHO. J Clin Med. (2023) 12:6275. doi: 10.3390/jcm12196275 37834919 PMC10573907

[69] Neelima P Gavarkovs A Tamez M Mattei J. The influence of diet on fertility and the implications for public health nutrition in the United States. Front Public Health. (2018) 6:211. doi: 10.3389/fpubh.2018.00211 30109221 PMC6079277

[70] Ahmad F Ahmed SH Choucair F Chouliaras S Awwad J Terranegra A. A disturbed communication between hypothalamic-pituitary-ovary axis and gut microbiota in female infertility: is diet to blame? J Trans Med. (2025) 23:92. doi: 10.1186/s12967-025-06117-x PMC11749209 39838491

[71] Zhuang Y Li LF Zhang YQ Liu XN Zeng BB Zhu BX. Association between life’s essential 8 and infertility as well as the mediating effects of oxidative stress and inflammatory factors among US women aged 18–45 years. Reprod Sci. (2024) 32:1–10. doi: 10.1007/s43032-024-01635-3 38977640

[72] Pan WY Ye XQ Zhu ZY Li CM Zhou JH Liu J. Urinary cadmium concentrations and risk of primary ovarian insufficiency in women: a case-control study. Environ Geochem Health. (2021) 43:2025–35. doi: 10.1007/s10653-020-00775-0 33222148

[73] Massányi P Massányi M Madeddu R Stawarz R Lukáč N. Effects of cadmium, lead, and mercury on the structure and function of reproductive organs. Toxics. (2020) 8:94. doi: 10.3390/toxics8040094 33137881 PMC7711607

[74] Mukherjee AG Wanjari UR Renu K Vellingiri B Gopalakrishnan AVa. Heavy metal and metalloid-induced reproductive toxicity. Environ Toxicol Pharmacol. (2022) 92:103859. doi: 10.1016/j.etap.2022.103859 35358731

[75] Manouchehri A Shokri S Pirhadi M Karimi M Abbaszadeh S Mirzaei G. The effects of toxic heavy metals lead, cadmium and copper on the epidemiology of male and female infertility. JBRA Assisted Reprod. (2022) 26:627. doi: 10.5935/1518-0557.20220013 PMC9635604 35916450

[76] Lee S Jin-young M Kyoung-bok M. Female infertility associated with blood lead and cadmium levels. Int J Environ Res Public Health. (2020) 17:1794. doi: 10.3390/ijerph17051794 32164251 PMC7084729

[77] Gonzalez-Martin R Palomar A Quiñonero A Pellicer N Fernandez-Saavedra R Conde-Vilda E. The impact of essential trace elements on ovarian response and reproductive outcomes following single euploid embryo transfer. Int J Mol Sci. (2023) 24:10968. doi: 10.3390/ijms241310968 37446146 PMC10341631

[78] Kek T Geršak K Kuželički NK Šturm DC Mazej D Tratnik JS. Associations of essential and non-essential trace elements’ Levels in the blood, serum, and urine in women with premature ovarian insufficiency. Biol Trace Element Res. (2025), 1–18. doi: 10.1007/s12011-024-04507-8 PMC12316735 39789351

[79] Abudawood M Alnuaim L Tabassum H Ghneim HK Alfhili MA Alanazi ST. An insight into the impact of serum tellurium, thallium, osmium and antimony on the antioxidant/redox status of PCOS patients: A comprehensive study. Int J Mol Sci. (2023) 24:3. doi: 10.3390/ijms24032596 PMC9917046 36768916

[80] Chi HB Tang JJ Fan XY Zhang HW Tang F Lin XS. Single-and combined-heavy metals/metalloids exposures are associated with infertility in US women aged 20-44: NHANES 2013–2020 analysis. Reprod Toxicol. (2025) 132:108851. doi: 10.1016/j.reprotox.2025.108851 39900206

[81] Palomar A Gonzalez-Martin R Quiñonero A Pellicer N Fernandez-Saavedra R Rucandio I. Bioaccumulation of non-essential trace elements detected in women’s follicular fluid, urine, and plasma is associated with poor reproductive outcomes following single euploid embryo transfer: A pilot study. Int J Mol Sci. (2023) 24:13147. doi: 10.3390/ijms241713147 37685954 PMC10487767

[82] Haile F Gebeyehu S Abdulkadir H Gizachew Y Hailet M. Determinants of infertility among married women who attend gynecologic unit at health facilities of Gamo Zone and South Omo Zone, Southern Ethiopia: a case control study. Contraception Reprod Med. (2025) 10:8. doi: 10.1186/s40834-024-00330-7 PMC11789352 39901287

[83] Khashayar P Pourghayoomi M Sharafi E Kashani L Shirzad N Hemmatabadiet M. Does prevalence of female sexual dysfunction differ among infertile patients with or without polycystic ovary syndrome: a cross-sectional study. Int J Fertil Steril. (2024) 18:367. doi: 10.22074/ijfs.2023.2005240.1486 39564828 PMC11589975

[84] Zeng H Chang YH Liu NH Li SY. Ectopic pregnancy is associated with increased risk of displaced implantation window: a retrospective study. BMC Pregnancy Childbirth. (2024) 24:839. doi: 10.1186/s12884-024-07072-z 39707276 PMC11661333

